# Interventions for managing clinically relevant sleep disturbances or insomnia in cancer patients and survivors: an up-to-date systematic review and meta-analysis of self-reported sleep disturbance

**DOI:** 10.3389/fpsyg.2026.1811748

**Published:** 2026-06-03

**Authors:** Julia Chan, Danielle Wing Lam Ng, Daniel Yee Tak Fong, Mathew Ching Hei Chan, Yijia Shi, Wendy Wing Tak Lam

**Affiliations:** 1 Li Ka Shing Faculty of Medicine, School of Public Health, Centre for Psycho-Oncology Research and Training, The University of Hong Kong, Hong Kong, Hong Kong SAR, China; 2 Li Ka Shing Faculty of Medicine, Jockey Club Institute of Cancer Care, The University of Hong Kong, Hong Kong, Hong Kong SAR, China; 3 Li Ka Shing Faculty of Medicine, School of Nursing, The University of Hong Kong, Hong Kong, Hong Kong SAR, China

**Keywords:** cancer survivorship, cognitive behavioural therapy for insomnia, complementary and alternative medicine, insomnia, sleep disturbance

## Abstract

**Background:**

Sleep disturbances are common after-effects of cancer. While the effectiveness of interventions in managing sleep disturbances in cancer have been evaluated, the effectiveness of interventions on reducing clinically relevant sleep disturbances remains unknown. This systematic review and meta-analysis aimed to appraise the effectiveness of up-to-date interventions managing clinical sleep disturbances in cancer patients or survivors.

**Methods:**

Data from randomized controlled trials were analysed following PRISMA guidelines. Five English databases were searched from January 2012 to June 2025. Outcomes were measured using Hedge’s g with random-effects models. The methodological quality of the trials was assessed by Cochrane Risk of Bias Tool 2.0 and the certainty of evidence was determined using GRADE.

**Results:**

42 trials with 3,844 participants were identified, with sample sizes ranging from 22 to 255. A moderate and large effect was found for cognitive behavioural therapy for insomnia (CBT-I) on improving sleep quality (*g* = 0.57; 95% CI = 0.20 - 0.93, *p* < 0.01) and insomnia severity (*g* = 0.91; 95% CI = 0.49 - 1.34, *p* < 0.01) respectively. Complementary and alternative medicine (CAM) showed a large effect size for improving sleep quality (*g* = 1.11; 95% CI = 0.43 - 1.78, *p* < 0.01) but indicated presence of publication bias. Other interventions, including mindfulness, exercise-based interventions, herbal medicine, relaxation and brief behavioural therapy for insomnia (BBT-I), showed less convincing evidence compared to CBT-I. Only one study scored low in overall risk of bias.

**Conclusion:**

The existing evidence base needs to be expanded to adequately evaluate the effectiveness of other interventions for clinical sleep disturbances. CBT-I is currently the most empirically supported treatment for cancer patients and survivors with clinically relevant sleep disturbances.

## Introduction

Sleep disturbance is common among cancer patients and negatively impacts their well-being and treatment outcomes, with a reported pooled prevalence of 60.7% (Al Maqbali et al., 2022). Disturbed sleep observed in patients with cancer is commonly characterized by difficulty in initiating sleep, maintaining sleep, and early morning awakening and daytime impairment (Berger, 2009), symptoms that closely resemble insomnia (Riemann et al., 2023). Sleep disturbances are commonly reported in oncology settings, and there is growing recognition that a substantial portion of cancer patients may meet the formal diagnostic criteria for insomnia disorder. As a result, insomnia remains underdiagnosed and undertreated amongst cancer patients and survivors (Pinucci et al., 2023). For example, there is evidence that 30% of cancer survivors continue to report unresolved sleep disturbances for up to two years following cancer treatment completion, and those classified as having “persistent high sleep disturbance” exhibited sleep characteristics consistent with the research diagnostic criteria for insomnia disorder (average sleep latency >30 min, wake after sleep onset >30 min, sleep efficiency <85%, and/or total sleep time <6.5 h) (Chan et al., 2023; Edinger et al., 2004; Lichstein et al., 2003). Cancer-related sleep disturbances are associated with detrimental consequences, including impaired cognitive functioning (Savard and Morin, 2001), reduced general quality of life (Liu et al., 2013), fatigue (Berger, 2009), and psychological distress (Mehnert et al., 2018). Persistent disruption of circadian rhythms may also facilitate cancer progression by perpetuating the dysregulation of inflammatory responses (Zhou et al., 2022). Additionally, cancer survivors may experience concerns about cancer recurrence due to poor sleep (Chan et al., 2024; Lowery-Allison et al., 2018).

Given the substantial burden of sleep disturbances in cancer patients and survivors, various interventions have been used to manage sleep disturbances. Short courses of pharmacological treatments for sleep disturbances are common in clinical practice, including hypnotics (benzodiazepines and non-benzodiazepines), antidepressants, anticonvulsants, and antihistamines (Pinucci et al., 2023). While well-studied in the general population (De Crescenzo et al., 2022), randomized controlled trials investigating their efficacy for managing sleep disturbance in cancer populations are limited. Amongst existing clinical trials, Theobald et al. (2002) found that the antidepressant mirtazapine did not significantly improve insomnia in patients with advanced cancer, while Tan et al. (2009) observed that olanzapine, an antipsychotic, improved insomnia in patients receiving chemotherapy. Although commonly prescribed for cancer patients, sleep medication can cause side effects such as dizziness, sedation, and impaired daytime functioning (Pinucci et al., 2023). Aside from the side effects, cancer survivors may refuse to take prescription aids because of concerns regarding drug dependency and tolerance (Reynolds-Cowie and Fleming, 2021). Alternatively, supplements such as melatonin, a hormone that regulates the sleep–wake cycle, have been used to improve sleep quality (Palmer et al., 2020). However, a recent meta-analysis did not find melatonin to significantly improve sleep quality in different cancer types (Fan et al., 2022). Valerian, an herbal supplement primarily sold as a sleeping aid, similarly has inconclusive evidence for improving sleep in a randomized controlled trial for cancer patients (Barton et al., 2011).

Considerably more empirical research has been published on non-pharmacological interventions for sleep disturbances in cancer patients. Due to compelling evidence of the effectiveness of cognitive behavioural therapy for insomnia (CBT-I) (Johnson et al., 2016; Squires et al., 2022), the National Comprehensive Cancer Network (NCCN) recommends CBT-I as the first-line treatment for insomnia in their survivorship guidelines (Denlinger et al., 2018). In contrast, other types of interventions have shown mixed results. A meta-analysis found moderate intensity walking exercises, alone or in combination with other interventions has improves sleep quality in those with cancer (Chiu, 2015). Aerobic and strength training have also shown small positive effects on subjective sleep quality and objective sleep onset latency (Fang et al., 2019). Likewise, Takemura et al. (2020) found that aerobic exercise and mind–body interventions, such as yoga and tai-chi, improve sleep outcomes in patients experiencing sleep disturbance. In contrast, a systematic review by Mercier et al. (2017) found aerobic, resistance, or flexibility exercises to positively impact sleep for patients with cancer, though their meta-analysis showed no statistically significant effects. Mindfulness-based stress reduction has also been found to significantly improve sleep quality in cancer survivors compared to usual care, but not when compared to active controls in a recent meta-analysis (Suh et al., 2021). Additionally, complementary and alternative medicine (CAM), such as acupuncture, acupressure and other approaches (e.g., reflexology, massage, footbath therapy) have shown mixed results in improving sleep in patients with cancer, which may be partially due to the quality of existing studies (Cheung et al., 2022; Choi et al., 2017; Guo et al., 2024; Weng et al., 2024; Kumar et al., 2024; Miladinia et al., 2017; Zengin and Aylaz, 2019).

Variation of previous findings can be attributed to several reasons. Existing reviews and meta-analyses have focused on specific intervention types for sleep disturbance such as CBTI-I (Johnson et al., 2016; Squires et al., 2022; Ma et al., 2021), exercise (Takemura et al., 2020; Mercier et al., 2017), or acupuncture (Weng et al., 2024; Liu et al., 2024; Yu et al., 2022), rather than evaluating the comparative effectiveness of different interventions. Additionally, published reviews often centre on specific types of cancer [e.g., lung (Papadopoulos et al., 2018), breast (Wong et al., 2022)] and included evidence beyond randomized controlled trials (Papadopoulos et al., 2018; Howell et al., 2014; Matthews et al., 2018), limiting generalizability for all cancer patients. A recently published meta-analysis assessed the various interventions for insomnia in cancer (Nissen et al., 2024) but excluded studies examining CAM. Given the increase of intervention types over the last decade, a comprehensive review of all types of interventions for cancer patients is needed.

Most importantly, prior reviews did not require participants of included studies to exhibit clinically relevant sleep disturbances, as determined by screening tools with an established clinical cut-off point [e.g., Insomnia Severity Index <7 (Bastien et al., 2001; Morin et al., 2011); Pittsburgh Sleep Quality Index <5 (Buysse et al., 1989)] or meeting diagnostic criteria for insomnia disorder [e.g., Diagnostic and Statistical Manual for Mental Disorders (American Psychiatric Association, 2013)]. For example, a meta-analysis examined the efficacy of exercise (Takemura et al., 2020) by primarily targeting studies with sample means meeting clinically relevant cut-offs, rather than individual participant criteria. Similarly, recent meta-analyses that have comprehensively evaluated the effectiveness of a variety of interventions for insomnia in cancer populations did not require all included studies to have an inclusion criterion for clinical sleep disturbance or insomnia (Nissen et al., 2024; Luo et al., 2024). Thus, findings may simply reflect general sleep quality improvement, rather than the effectiveness of reducing clinically relevant sleep disturbances or insomnia.

Given the absence of standardized guidelines for managing sleep disturbances in cancer patients and the increasing variety of intervention methods available, this systematic review and meta-analysis aim to evaluate the effectiveness of existing interventions specifically designed to address clinically relevant sleep disturbances or insomnia in a diverse population of cancer patients and survivors.

## Methods

Methods of the systematic review and meta-analysis adhered to the guidelines set forth by the Preferred Reporting Items for Systematic Reviews and Meta-Analyses (PRISMA) Statement (Page et al., 2021). The protocol has been registered with PROSPERO, the international prospective register for systematic reviews, under the ID CRD42023469727.

### Eligibility criteria for studies

Studies were selected according to the following criteria: population, interventions, comparators, outcomes (PICO) and types of studies.

#### Population

The studies included adults aged 18 years or older with cancer at any stage of the cancer care trajectory (before, during, or after anti-cancer treatment) who presented with clinical levels of sleep disturbance or insomnia screened with a valid tools with clinical cut-off scores for sleep disturbance [e.g., Insomnia Severity Index (ISI), Pittsburgh Sleep Quality Index (PSQI)] or diagnostic criteria for insomnia (e.g., The Diagnostic and Statistical Manual of Mental Disorders) during recruitment. No pre-specified cut-off was required for inclusion, as various validated instruments with varying clinical thresholds have been used to define poor sleep quality or insomnia in cancer patients or survivors. The clinical practice guidelines issued by the European Society for Medical Oncology (ESMO) have underscored the lack of a standardised instrument or method for the assessment of insomnia in cancer patients (Grassi et al., 2023). For example, a recent meta-analysis found that 59 studies utilized different PSQI cut-off scores to identify poor sleep, ranging from ≥5 to ≥10, across the literature examining cancer patients (Chen et al., 2024). Studies that focused on adolescents, young adults, survivors of childhood cancer, solely advanced cancer patients (e.g., patients with metastatic cancer or only stage 4 and above) or caregivers of cancer patients were excluded.

#### Intervention

Pharmacological and non-pharmacological intervention studies targeting sleep disturbance, offered in any setting (home, hospital, or rehabilitation centre) were included. Pharmacological interventions include oral medication or supplements, including prescription based (benzodiazepines, benzodiazepine receptor agonists, non-benzodiazepines, antidepressants, antihistamines, anticonvulsants, melatonin agonist, antipsychotics), non-prescription based dietary supplements (e.g., melatonin, valerian root). Orally administered herbal or Traditional Chinese medicine (TCM) was also included. Non-pharmacological interventions included psychological/behavioural interventions (e.g., cognitive behavioural therapy for insomnia, sleep restriction, relaxation, mindfulness based intervention, expressive writing, brief-behavioural therapy for insomnia), education (e.g., sleep hygiene), exercise [e.g., resistance training, walking, yoga, qigong, tai-chi as well as mind–body exercises such as yoga, qigong, tai chi as it combines body movement with a focus on breathing, a calm state or mind and deep states or relaxation (Larkey et al., 2009) and has been typically examined collectively with physical exercise (Duan et al., 2020; Kreutz et al., 2019)], dietary (e.g., weight-loss), multimodal (e.g., combination of interventions, nursing, remote monitoring) and bright light therapy, as well as complementary and alternative medicine (CAM) (e.g., aromatherapy, massages, foot reflexology, homeopathy, moxibustion, music therapy, acupuncture, acupressure, hypnosis) and biofeedback therapy (e.g., heart rate variability, neurofeedback) as well as electrical stimulation (e.g., cranial electrical stimulation). No restrictions were set on the duration of the intervention or follow-up time. Stepped-care interventions were excluded since participants may undergo different interventions even at the first step, depending on symptom severity (e.g., participants with ISI score ≥ 8 to < 15 and those ≥ 15), thus limiting the ability to make meaningful comparisons of sleep disturbance outcomes across groups at post-intervention (Savard et al., 2021; Garneau et al., 2024).

#### Comparator

The comparators of the intervention were passive controls (e.g., no intervention, usual care or waitlist control), active controls (e.g., attentional control, sleep hygiene), as well as sham/placebo and competing interventions (e.g., CBT-I versus exercise).

#### Outcome

The primary outcome of the review was sleep disturbance. Studies with measures with feasibility or acceptability (e.g., recruitment, attrition, adherence, satisfaction) as a primary objective or primary outcome were excluded, as these trials are not designed to detect meaningful treatment effects. Due to typically small sample sizes, feasibility studies often yield imprecise effect estimates, as they are not formally powered to achieve adequate statistical precision (Sim, 2019; Teresi et al., 2022).

Sleep disturbance should be assessed using an established measure or subscale within an established self-report instrument such as the Insomnia Severity Index (ISI) (Bastien et al., 2001; Morin et al., 2011), Pittsburgh Sleep Quality Index (PSQI) (Buysse et al., 1989) and Athens Insomnia Scale (AIS) (Soldatos et al., 2000). As single-item or unidimensional assessments may not exhibit the same level of sensitivity or specificity in detecting sleep disturbance or insomnia in cancer (Passik et al., 2003), only validated multi-item scales were included, and single-item or unidimensional assessments [e.g., sleep item in the European Organization For Research And Treatment Of Cancer Core Quality of Life Questionnaire (EORTC-QLQ-30) (Fayers et al., 2002), sleep duration from the sleep diary (Carney et al., 2012)] were excluded unless they had a multi-item sleep disturbance scale as a supplementary or secondary outcome. Studies should report outcomes as means and standard deviations (SD) or standard errors (SE) in all intervention groups or provide data convertible into an effect size (ES). Studies relying solely on objective sleep assessment methods such as actigraphy were excluded due to variability in device models (Madsen et al., 2015) and inaccuracies in measuring sleep patterns (Vallières and Morin, 2003). Data was extracted at post-intervention, as well as at follow-up assessments where available. Follow-up assessments were categorized as short-term follow-up if conducted within 3 months post-intervention, and as long-term follow-up if the assessment extended beyond 3 months post-intervention.

#### Types of studies

This review only considered randomized controlled trials with random assignments to treatments or control groups. Non-randomized controlled trials, pre-post studies, case series, case reports, reviews, comments, letters, descriptive papers, observational studies, meta-analyses and systematic reviews were excluded. Only full-text articles published in English were considered.

### Information sources and search strategy

Keyword-based searches were conducted in PubMed, PsycINFO, Embase, Web of Science and Cochrane Central Register of Controlled Trials (CENTRAL) databases. "Cancer" and "neoplasm" were combined with keywords related to intervention (e.g., "therapy", "treatment", "programme") and terms related to sleep disturbance (e.g., "sleep", "sleep quality", "insomnia). Equivalent MeSH terms were used in conjunction with the listed keywords for the PubMed database (Supplementary Table 1). The keyword "randomized controlled trial" was combined with the above-mentioned keywords, unless a filter for randomized controlled trials was available. Reference lists of existing systematic reviews and meta-analyses for sleep disturbance interventions for cancer patients were checked to identify any relevant studies that were unidentified by the initial search.

Considering that research on sleep disturbance in cancer is relatively new as the first population-based prevalence study reporting on sleep disturbances (Davidson et al., 2002) and the first randomized controlled trial on sleep disturbance interventions for cancer patients (Epstein, 2007) was conducted within two decades ago, intervention studies conducted earlier may not reflect the current state of cancer treatment and may not be relevant to the current cancer population. Therefore, studies published within the previous ten years of when the literature search was conducted (2012- September 2022) were imposed. A search update was conducted in January 2024 to include studies up to December 2023. A second search update was conducted in July 2025, to include studies from January 2024 up to June 2025.

### Study screening and data extraction

The searched articles were imported into the reference manager software Endnote 20 (Clarivate Analytics, PA, USA) with duplicates removed. Four reviewers (J.C., J.K.K.L., Y.S., M.C.H.C.) completed the title and abstract screening and full-text screening in a blinded standardized manner against the inclusion criteria. A fifth review author (D.W.L.N.) resolved the discrepancies by consensus or discussion. Data was extracted by J.C. into Windows Excel. The details extracted included study characteristics (authors, publication year), participant characteristics (sample size, cancer type, mean age), screening condition (clinical cut-off or diagnostic criteria used for sleep disturbance), intervention details (type of intervention, setting, comparator), and outcomes (instrument for sleep disturbance) as well as raw outcome data (mean and standard deviation/standard error).

### Assessment of risk of bias and certainty of evidence

Risk of bias was assessed using the Cochrane Handbook’s “Risk of Bias 2” tool, to assess bias from the randomization process, bias due to deviations from the intended interventions, bias from missing outcome data, bias in the measurement of the outcome, and bias in the selection of the reported result. Studies with serious concerns related to the risk of bias were considered high risk and those with limited reporting were considered unclear risk. The assessment was performed independently by two review authors (J.C. and D.W.L.N.); and disagreements were resolved by consensus or discussion.

Certainty of evidence was assessed using the Grading of Recommendations Assessment Development and Evaluations (GRADE) approach for domains of risk of bias, inconsistency, indirectness, imprecision, and publication bias in all outcomes (including post-treatment and follow-up periods) per intervention type (Guyatt et al., 2008). It was conducted using the online software GRADEpro GDT by two independent reviewers (J.C. & D.W.L.N.) and disagreements were resolved by consensus or discussion. The certainty of the evidence was graded as “very low”, “low”, “moderate,” or “high”.

### Statistical analysis

The between-group ESs were calculated using Hedge’s g, a variation of Cohen’s d, which corrects for possible bias due to the small sample sizes (Hedges and Olkin, 2014). Whenever possible, ESs were computed using means and standard deviations. In cases where missing or inconsistent data was present in the relevant studies, data were requested from the corresponding authors by email; otherwise, ESs were estimated based on other reported statistics, such as standard errors, within-group or between-group *p*-values, *F*-values, and Cohen’s d, according to the Cochrane Handbook for Systematic Reviews of Interventions (Deeks et al., 2024).

Due to substantial clinical and methodological heterogeneity across studies (different cancer types, mixed cancer stages, cancer treatment status, sleep disturbance instruments, clinical sleep disturbance requirements, and intervention delivery formats), limited direct comparisons between intervention types, and fragmented connectivity within the network, a full network meta-analysis was deemed infeasible (Ades et al., 2024; Florez et al., 2024; Phillips et al., 2022). Pairwise meta-analyses were conducted in cases with sufficient studies (K) (K ≥ 2) pertaining to each intervention type and outcome measure (Deeks et al., 2024). ESs were pooled and weighed using the calculated inverse standard error. A random-effects model was chosen for all analyses with positive values indicating ESs in the hypothesized direction. Heterogeneity was assessed using Cochrane’s Chi-square test (Q test) to assess significant heterogeneity among the ES estimates and I^2^ tests to quantify the degree of heterogeneity of the included studies. Meta-analyses were also conducted on follow-up assessments where available. The most common follow-up time points or those closest in duration were selected for short-term (≤3 months post-intervention) or long-term follow-up (>3 months post-intervention) meta-analyses. The 3 months post-intervention mark was chosen as the threshold to distinguish between short- and long-term follow-up, as it represented the most frequently reported assessment time point across the included studies.

In instances of multi-arm studies, several approaches were used to examine relevant comparisons while overcoming a unit-of-analysis error or “double-counting” (Axon et al., 2023). Given that the aim of the meta-analysis was to examine the effectiveness of different types of interventions, intervention arms that were conceptually similar (e.g., Mind–body Bridging versus Mindfulness Meditation) were combined. If a study had both a competing intervention arm as well as a control arm (e.g., acupressure, mindfulness, passive control), the sample size (*N*) of the shared intervention group would be split to avoid “double counting” (e.g., split acupressure *N* versus mindfulness or control). Similarly, in studies where both passive and active control arms were used (e.g., waitlist control, attentional control), the shared intervention group *N* was split. In studies where multiple intervention arms were used to investigate the impact of delivery format (e.g., online CBT-I versus face-to-face CBT-I), the control group *N* would be split. Intervention arms that included a mix of conceptually different components (e.g., mindfulness combined with acupressure) were omitted from the analysis.

The possibility of publication bias was evaluated using funnel plots and Egger’s test (Egger et al., 1997) when K ≥ 10. If the results were suggestive of publication bias, the Duval and Tweedie trim-and-fill method was used to produce an adjusted ES (Duval and Tweedie, 2000). Sensitivity analyses were conducted to include feasibility studies that had been excluded during the selection process to assess their potential influence on the pooled ES and publication bias.

Sources of heterogeneity were explored post-hoc by conducting subgroup analyses when the meta-analyses contained at least 10 studies (K ≥ 10) (Deeks et al., 2024). Subgroup analyses were performed according to intervention characteristics (e.g., delivery, format), study characteristics (e.g., comparator types), participant characteristics (cancer type, period of cancer treatment, age, permitted use of sleep medication) and risk of bias (some or low versus high).

Post-hoc analyses were conducted to examine remission rates of insomnia or poor sleep for intervention types that demonstrated statistically significant effects at post-intervention in the meta-analysis. To ensure consistency, only studies that reported remission rates using the same thresholds applied during participant screening were included in this analysis. For instance, if a study enrolled participants with an Insomnia Severity Index (ISI) score of ≥8, remission was defined as achieving an ISI score below 8 at post-intervention.

ES calculations were performed using Windows Excel, Cochrane’s RevMan calculator and the software R using the package “esc.” Meta-analyses, forest plots and post-hoc analyses, including Egger’s test and trim-and-fill procedures were produced using packages “esc,” “metafor” and “meta” (computer data code can be accessed in Supplementary material 1).

## Results

**Figure 1:**
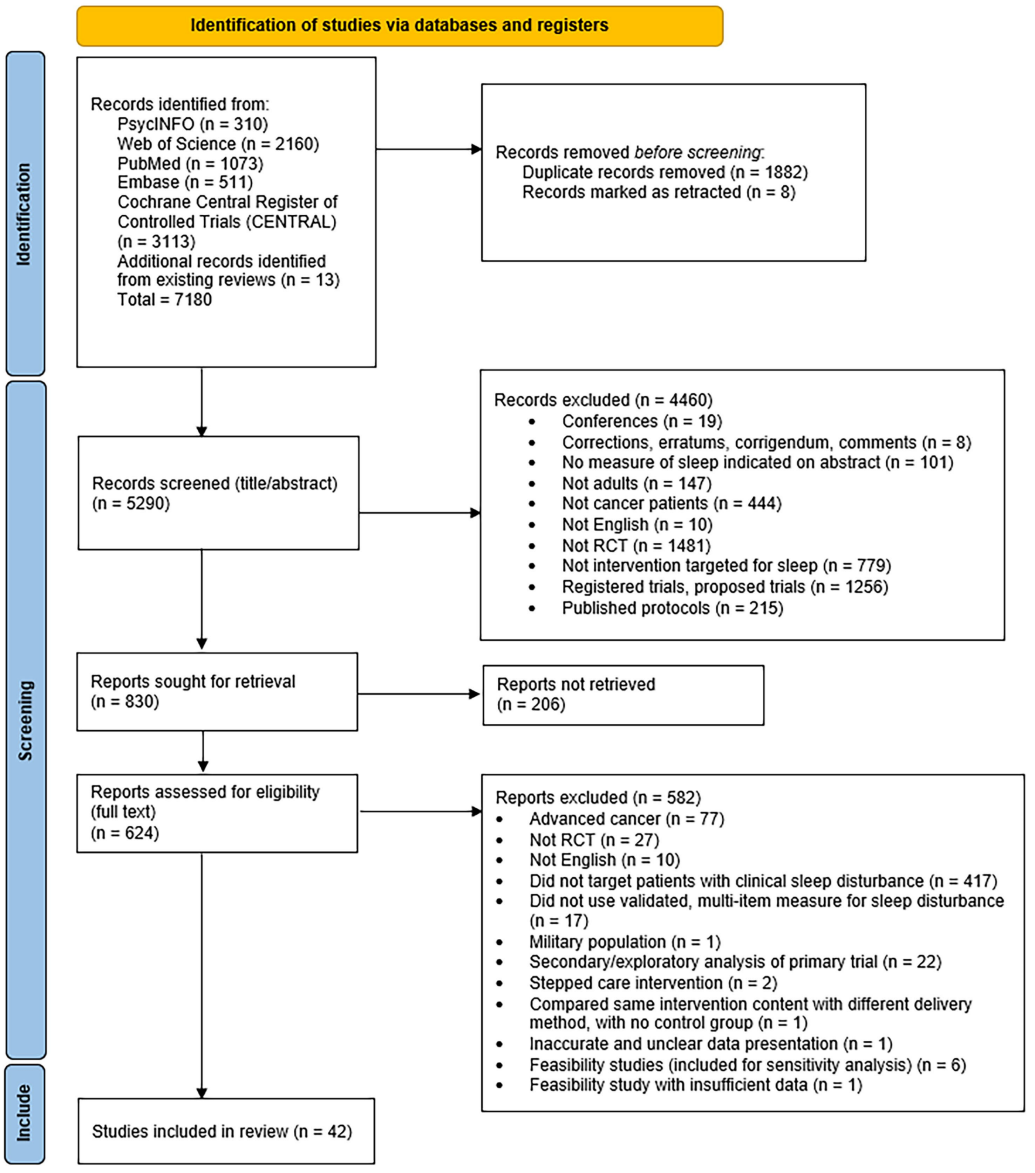
PRISMA flow diagram.

The literature search yielded 7,180 results. After removing duplicates, 5,290 records underwent title and abstract screening, and 624 records underwent full-text screening. After screening, 42 studies were identified to be eligible for the systematic review and meta-analysis (Miladinia et al., 2017; Zengin and Aylaz, 2019; Alem et al., 2021; Barton et al., 2020; Bi et al., 2024; Casault et al., 2015; Chung et al., 2022; Clara et al., 2025; Dean et al., 2020; Dickerson et al., 2024; Garland et al., 2014; Garland et al., 2024; Garland et al., 2019; Guo et al., 2025; Hamzeh et al., 2020; Hoextermann et al., 2021; Irwin et al., 2017; Kahreh et al., 2024; Kuo et al., 2018; Lee et al., 2018; Li et al., 2024; Liu et al., 2022; Matthews et al., 2014; Mercier et al., 2018; Moon et al., 2020; Myers et al., 2025; Nakamura et al., 2013; Nourizadeh et al., 2022; Ritterband et al., 2012; Sarı et al., 2024; Savard et al., 2014; Shahrokhi et al., 2021; Tian et al., 2025; Turan et al., 2024; Turan et al., 2023; Wang et al., 2022; Yoon and Park, 2019; Zachariae et al., 2018; Zhang et al., 2019; Zhang et al., 2023; Zhang et al., 2021; Zhao et al., 2020). The study selection process is illustrated in Figure 1.

### Study and participant characteristics (Table 1)

**Table 1:** Study and participant characteristics.

No.	Study (year)	Country	Cancer type	Cancer staging	Cancer treatment status	Allow use of sleep medication	Mean age	Intervention type; intervention(s)	Comparator type; Comparator	Clinical SD requirement	SD outcome (s)	Follow-up assessments	Total randomised *N*; total analysed *N* at post-intervention	Risk of bias
1	Alem et al. (2021)	Iran	Mixed	Not reported	Undergoing treatment	Yes	52.3	CBT-I; sleep training with CBT-I components	Passive; routine care	PSQI ≥ 5	PSQI	One week, three weeks post-intervention	70; 70	High
2	Barton et al. (2020)	USA	Mixed	Stage I-IV	Completed primary treatment	No	Not reported	CBT-I; face-to-face	Active; sleep hygiene	Diagnostic criteria for insomnia	PSQI	None	81; 67	High
3	Bi et al. (2024)	China	Mixed	Stage I-III	Undergoing treatment	No	47.61	CAM; Auricular Acupressure, Positive psychological intervention, Auricular acupressure combined with Positive psychological intervention	Placebo; not required to press the acupoints	PSQI ≥ 8	PSQI	1-month post-intervention	120; 109	Some concerns
4	Casault et al. (2015)	Canada	Mixed	Non-metastatic	Not completed primary treatment; percentage of patients undergoing primary treatment	No	56.9	CBT-I; self-help CBT-I, with biweekly phone calls from psychologist	Passive; no intervention	ISI ≥ 8 and clinical interview for insomnia (Insomnia Interview Schedule)	ISI	3 months, 6 months post-intervention	38; 32	High
5	Chung et al. (2022)	South Korea	Mixed	Stage I-IV	Not reported	No	47.53	CBT-I; digital CBT-I	Active; attentional control, Passive; waitlist	PSQI ≥ 8.5	PSQI	None	57; 36	High
6	Clara et al. (2025)	Portugal	Mixed	Survivors	Completed primary treatment	Yes	47	CBT-I; web-based CBT-I	Passive; waitlist	ISI ≥ 8	ISI	None	154; 138	High
7	Dean et al. (2020)	USA	Lung	Stage I-II	Not completed primary treatment; ≥ 6 weeks from surgical tumour removal	Not reported	65.63	BBT-I	Active; healthy eating	ISI ≥ 8 and diagnostic criteria for insomnia	ISI/PSQI	None	40; 30	Some concerns
8	Dickerson et al. (2024)	USA	Mixed	Survivors	Completed primary treatment	Not reported	63.7	BBT-I	Active; healthy eating	ISI ≥ 8	ISI/PSQI	3 months, 12 months post-intervention	132; 122	Some concerns
9	Garland et al. (2014)	Canada	Mixed	Non-metastatic	Completed primary treatment	Yes, if dose is stable	58.89	Mindfulness; mindfulness based stress reduction (MBSR)	Competing; CBT-I	Diagnostic criteria for insomnia	ISI/PSQI	3 months post-intervention	111; 72	Low
10	Garland et al. (2019)	USA	Mixed	Stage I-IV	Completed primary treatment	Yes, if dose is stable	61.5	CAM; acupuncture	Competing; CBT-I	ISI ≥ 8 and diagnostic criteria for insomnia	ISI/PSQI	1 month, 2 months, 3 months post-intervention	160; 148	High
11	Garland et al. (2024)	USA	Mixed	Stage 0-IV	Completed primary treatment	Yes, if dose is stable	60.23	CBTI; virtual CBT-I	Passive; waitlist	ISI ≥ 8 and diagnostic criteria for insomnia	ISI/PSQI item 6	None for sleep disturbance (follow-up for cognitive impairment)	132; 121	High
12	Guo et al. (2025)	China	Breast	Not reported	Undergoing treatment	No	51.6	Relaxation; Benson relaxation technique (BRT), Dynamic and static relaxation therapy	Passive; routine care	PSQI ≥ 8	PSQI	None	114; 109	High
13	Hamzeh et al. (2020)	Iran	Mixed	Not reported	Not reported	No	49.47	CAM; Lavender essential oil, peppermint essential oil	Placebo; distilled water was mixed with 1% essential oil	PSQI ≥ 5	PSQI	None	120; 120	Some concerns
14	Hoextermann et al. (2021)	Germany	Breast	Stage I-III	Completed primary treatment	No	55.73	CAM; auricular acupuncture	Active; psychoeducation	Diagnostic criteria for insomnia	PSQI	3 months, 6 months post-intervention	52; 43	Some concerns
15	(2017) Irwin et al. (2017)	USA	Breast	Survivors	Completed primary treatment	Yes	59.8	Exercise; Tai Chi Chih (TCC)	Competing; CBT-I	Diagnostic criteria for insomnia	AISI/PSQI	3 months, 12 months post-intervention	90; 80	Some concerns
16	Kahreh et al. (2024)	Iran	Mixed	Not reported	Undergoing treatment	Yes	45.95	Relaxation; progressive muscle relaxation	Passive; no intervention	PSQI ≥ 5	PSQI	None	70; 90	High
17	Kuo et al. (2018)	Taiwan	Gynaecological	Stage I-IV	Undergoing treatment	Yes	51.5	CAM; auricular point acupressure (APA)	Active; verbal and written advice on sleep hygiene	PSQI > 5	PSQI	None	47; 40	High
18	Lee et al. (2018)	South Korea	Mixed	Stage I-IV	Not completed primary treatment; percentage of patients undergoing primary treatment	Yes, if dose is stable	54.15	Herbal medicine; Gamiguibi-tang	Passive; waitlist	PSQI > 5	ISI	None	30; 26	High
19	Li et al. (2024)	China	Colorectal	Stage I-IV	Not reported	No	61.44	Behavioural activation (BA)	Passive; usual care	PSQI > 5	PSQI	None	113; 101	High
20	Liu et al. (2022)	China	Breast	Stage I-IV	Undergoing treatment	No	51.41	CAM; acupressure, Mindfulness; mindfulness-based stress reduction (MBSR), Mindfulness combined with CAM; MBSR combined with acupressure	Passive; routine care	PSQI ≥ 8 or more & sleep efficiency of less than 85% according to wrist actigraphy	PSQI	None	147; 134	High
21	Matthews et al. (2014)	USA	Breast	Stage I-III	Completed primary treatment	Yes, if dose is stable	51.51	CBT-I; face-to-face	Active; behavioural placebo treatment	ISI ≥ 8, diagnostic criteria for insomnia & clinical interview	ISI	3 months, 6 months post-intervention	60; 56	Some concerns
22	Mercier et al. (2018)	Canada	Mixed	Stage I-IV	Completed primary treatment	Yes	57.1	Exercise; home-based aerobic exericse	Competing; CBT-I (self-administered, with weekly phone call from researcher)	ISI ≥ 8	ISI/PSQI	3 months, 6 months post-intervention	41; 38	Some concerns
23	Miladinia et al. (2017)	Iran	Leukemia	Stage I-IV	Undergoing treatment	Not reported	34.5	CAM; slow-stroke back massage (SSBM)	Passive; routine care	PSQI > 5	PSQI	None	64; 60	High
24	Moon et al. (2020)	South Korea	Mixed	Stage I-IV	Completed primary treatment	No	63	Herbal medicine; Cheonwangbosimdan	Competing; CBT-I	ISI ≥ 8 and diagnostic criteria for insomnia	ISI/PSQI	None	22; 22	High
25	Myers et al. (2025)	USA	Prostate	Stage I-IV	Completed primary treatment	Yes	69.09	CBTI; Telehealth-delivered CBT-I with Readiwatch	Active; Readiwatch	ISI ≥ 8	ISI/PSQI/ PROMIS Sleep Disturbance	None	45; 40	
26	Nakamura et al. (2013)	USA	Mixed	Stage I-IV	Completed primary treatment	Yes	52.6	Mindfulness; mind–body bridging program (MBB), mindfulness meditation (MM)	Active; sleep hygiene	MOS-S ≥ 35	MOS-SS	1-month post-intervention	57; 55	High
27	Nourizadeh et al. (2022)	Iran	Breast	Stage II-III	Completed primary treatment	No	38.4	CAM; self-acupressure, Exercise; aerobic exercise training	Passive; routine care	PSQI ≥ 5	PSQI	None	99; 99	High
28	Ritterband et al. (2012)	USA	Mixed	Stage I-IV	Completed primary treatment	Yes, if dose is stable	56.7	CBT-I; SHUTi internet intervention based on CBT-I	Passive; waitlist	Diagnostic criteria for insomnia	ISI	None	28; 28	High
29	Sarı et al. (2024)	Turkey	Mixed	Stage II-III	Undergoing treatment	No	53.5	Relaxation; progressive muscle relaxation	Passive; no intervention	PSQI ≥ 5	PSQI	None	80; 69	High
30	Savard et al. (2014)	Canada	Breast	Stage I-III	Completed primary treatment	Yes, part of inclusion criteria	54.4	CBT-I; face-to-face, CBT-I; self-help and video-based CBT-I with option to call psychologist	Passive; no intervention	ISI ≥ 8 and use of sleep medication for 2 weeks or more	ISI	None	242; 204	High
31	Shahrokhi et al. (2021)	Iran	Colorectal	Not reported	Undergoing treatment	No	63.86	Pharmacotherapy; Zolpidem	Competing; melatonin	PSQI ≥ 5	PSQI	1-month post-intervention	101; 90	High
32	Tian et al. (2025)	China	Mixed	Survivors	Completed primary treatment	No	50.83	Transcranial magnetic stimulation (rTMS)	Competing; CBT-I, Placebo; sham transcranial magnetic stimulation	ISI ≥ 14, PSQI ≥ 8, and diagnostic criteria for insomnia	ISI/PSQI	None	66; 46	Some concerns
33	Turan et al. (2023)	Turkey	Colon and Pancreatic	Stage II-III	Undergoing treatment	Yes	58.81	CAM; self-acupressure	Passive; no intervention	PSQI > 5	PSQI	None	60; 60	High
34	Turan et al. (2024)	Turkey	Mixed	Stage I-IV	Undergoing treatment	Yes	61.61	Relaxation; progressive muscle relaxation	Passive; no intervention	PSQI > 5	PSQI	None	74; 74	High
35	Wang et al. (2022)	China	Breast	Stage I-IV	Undergoing treatment	Yes	53.47	CAM; adaptive-auricular point acupressure (APA)	Active; verbal and written advice on sleep hygiene	PSQI > 7	PSQI	None	76; 68	High
36	Yoon and Park (2019)	South Korea	Breast	Stage I-III	Undergoing treatment	Yes	44.81	CAM; auricular acupressure	Placebo; acupoints not indicated for insomnia	ISI ≥ 8	PSQI	None	46; 41	High
37	Zachariae et al. (2018)	Denmark	Breast	Stage I-III	Not completed primary treatment; surgically treated	Yes	50.2	CBT-I; SHUTi internet intervention based on CBT-I	Passive; waitlist	PSQI > 5	ISI/PSQI	1.5 months post intervention	255; 203	High
38	Zengin and Aylaz (2019)	Turkey	Mixed	Stage I-IV	Undergoing treatment	No	Not reported	CAM; sleep hygiene education and reflexology	Passive; no intervention	PSQI ≥ 5	PSQI	None	176; 167	High
39	Zhang et al. (2019)	China	Gynaecological	Stage I-III	Completed primary treatment	Yes, if using stable dose of hypnotics, not psychotropics	58.12	Mindfulness; mindfulness based stress reduction (MBSR)	Passive; usual care	Diagnostic criteria for insomnia	ISI	6 months, 12 months post-intervention	70; 64	Some concerns
40	Zhang et al. (2021)	Hong Kong SAR China	Breast	Stage I-IV	Not completed primary treatment; undergoing or have completed chemotherapy ≤6 months	Yes	52.6	CAM; electroacupuncture plus auricular acupressure	Passive; waitlist	ISI ≥ 10 and diagnostic criteria for insomnia	ISI/PSQI	1 month, 2 months post-intervention	30; 29	High
41	Zhang et al. (2023)	Hong Kong SAR China	Breast	Stage I-IV	Not completed primary treatment; underwent or had completed chemotherapy no more than 6 months	Yes	52.2	CAM; acupuncture	Placebo; acupoints that are not indicated for insomnia	ISI ≥ 10 and diagnostic criteria for insomnia	ISI/PSQI	3 months, 6 months post-intervention	138; 117	Some concerns
42	Zhao et al. (2020)	China	Breast	Stage I-III	Completed primary treatment	Yes	53.04	Mindfulness; mindfulness-based cognitive therapy for insomnia (MBCT-I)	Passive; waitlist	Diagnostic criteria for insomnia	ISI	3 months, 6 months post-intervention	136; 132	High

In studies where CBT-I served as the competing comparator (Garland et al., 2024; Garland et al., 2019; Irwin et al., 2017; Mercier et al., 2018; Moon et al., 2020; Tian et al., 2025), calculated effect sizes were reversed to reflect the effect of CBT-I relative to the alternate intervention in order to match the grouping of the intervention type. SD = sleep disturbance, ISI=Insomnia Severity Index, AISI = Athens Insomnia Index, PSQI=Pittsburgh Sleep Quality Index, MOS-S = Medical Outcomes Study Sleep Scale, CBT-I=Cognitive Behavioural Therapy for Insomnia, BBTI=Brief Behavioural Therapy for Insomnia, CAM = Complementary and Alternative Medicine; PROMIS=Patient-Reported Outcomes Measurement Information System.

The 42 randomized controlled trials had randomized a total of 3,844 cancer patients or survivors. The sample sizes ranged from 22 to 255, with a median of 75. The studies were conducted in the United States of America (K = 10), Iran (K = 6), Mainland China (K = 8), Canada (K = 4), South Korea (K = 4), Turkey (K = 4), Hong Kong (K = 2), Taiwan (K = 1), Denmark (K = 1), Portugal (K = 1) and Germany (K = 1).

Most of the studies were conducted on patients with mixed type cancer (K = 21) or patients with breast cancer (K = 13). Two studies examined gynaecological cancer, two studies examined colorectal cancer, and four studies examined lung, leukaemia, colon & pancreatic and prostate cancers, respectively. Most studies examined patients with mixed stages (stages I to IV) (K = 19) or early-stage cancer (stages I to III, survivors, non-metastatic) (K = 18), and five studies did not explicitly state the cancer staging of their participants. No studies have solely examined patients with advanced cancer as according to our inclusion criteria. 75.9% of the participants were female, given the large number of studies examining patients with breast cancer. The mean age of the participants was 54.3, excepting two studies that did not report this. The majority of studies examined participants who had completed primary cancer treatment (K = 19) or undergoing treatment (K = 14). Six studies were classified as not having completed primary treatment (e.g., undergoing or had completed adjuvant treatment). Three studies did not explicitly state the cancer treatment status. More than half of the included studies permitted participants to use sleep medications (K = 24).

Interventions examined included cognitive behavioural therapy for insomnia (CBT-I) (K = 17), complementary and alternative medicine (CAM) (K = 14), which included acupressure (K = 7), acupuncture (K = 3), electroacupuncture plus acupressure (K = 1), slow-stroke back massage (K = 1), reflexology (*n* = 1) and essential oils (K = 1). Other intervention types included mindfulness (K = 5), exercise (including one trial examining tai-chi) (K = 3), relaxation (K = 4), brief behavioural therapy for insomnia (K = 2) and herbal medicine (K = 2). Four studies compared the intervention effects of melatonin and zolpidem, behavioural activation, psychological positive intervention and transcranial magnetic stimulation, respectively. Two studies examined the combined interventions, one evaluated the effect of mindfulness-based stress reduction with acupressure, while the other explored the combined effect of positive psychological intervention and acupressure, respectively. Control conditions included passive control (e.g., no intervention, waitlist control, routine care; K = 21), active controls (e.g., sleep hygiene, psychoeducation and healthy eating, behavioural placebo; K = 10) and placebo (e.g., acupoints not indicated for acupuncture) (K = 5).

While herbal medicine has been previously categorised under the broader umbrella of CAM, the mode of administration of herbal medicine differs from acupressure, acupuncture, massage, reflexology, and essential oils. Herbal medicines are ingested orally, whereas the latter are applied externally. Previous research examining CAM use in cancer patients have also referred to herbal medicine as separate therapeutic approaches, considering herbal medicine as “biologically based therapies” (Balneaves et al., 2018; Drozdoff et al., 2018). As such, the effect of herbal medicine was analysed separately from other CAM modalities in the meta-analysis.

The majority of studies utilized the validated sleep disturbance outcome measures of the PSQI (K = 32) and ISI (K = 20) to measure sleep quality and insomnia severity, respectively. Additionally, two studies used the Medical Outcomes Study Sleep Scale (MOS-S) and the Athens Insomnia Severity Index (AISI) respectively. Given that MOS-S is conceptually similar to the PSQI, as they both assess overall sleep quality, the study using MOS-S was grouped with those using PSQI in the meta-analyses. Meanwhile, the study using AISI was grouped with those using the ISI for the same reasons. One study used both the ISI and item 6 of the PSQI (subjective sleep quality). Since the global PSQI score was not assessed in this study, only the ISI was analysed. One study utilised three validated measures, including the ISI, PSQI, and the PROMIS (Patient-Reported Outcomes Information System) sleep disturbance scale. Since PSQI and ISI were the most commonly used across studies, and the PROMIS sleep disturbance scale was unique to this single study, only the ISI and PSQI outcomes were retained for the analysis to ensure consistency and comparability.

The included studies utilized a variety of methods to select participants with clinically relevant sleep disturbances or insomnia. Seven studies utilized a diagnostic criteria for insomnia disorder, seven required participants to score the PSQI greater than 5, seven required scoring the PSQI of 5 or more, two required scoring the PSQI of 8 or more, six utilized a combination of ISI of 8 or greater and the use of a diagnostic criteria or screening interview for insomnia, five required the ISI of 8 or greater, and two used a combination of meeting the ISI of 10 or above and a diagnostic criteria for insomnia disorder. Six studies had standalone requirements: (1) scoring the PSQI greater than 7, (2) PSQI of 8.5 or more, (3) a combination of ISI of 8 or more and sleep efficiency of less than 85% according to the wrist actigraphy, (4) ISI of 8 or greater and use of sleep medication for 2 weeks or more, (5) a combination of ISI of 14 or more, PSQI of 8 or more, and a diagnostic criteria for insomnia disorder, and (6) scoring on the MOS-S of 35 or more.

### Effectiveness of different intervention types managing in relevant sleep disturbances or insomnia

The results of the meta-analyses of different intervention types according to outcome measures are shown in Table 2.

**Table 2:** Meta-analyses results for different intervention types according to sleep disturbance outcomes at post-intervention.

Intervention type	Heterogeneity							Effect size (ES)			Publication bias; Egger’s test		Adjusted ES; trim-and-fill		
Sleep outcome (measure)	K	Comparisons	N	I^2^	T^2^	Q (df)	*p*-value	g	95% CI	*p*-value	t (df)	*p*-value	g	95% CI	*p*-value
CBT-I															
Insomnia severity (ISI/AISI)	14	16	1,228	88.8	0.55	134.26 (15)	<0.01	0.91	0.49–1.34	**<0.01**	−0.41 (14)	0.69	-		
Sleep quality (PSQI)	11	13	831	73.1	0.29	44.56 (12)	<0.01	0.57	0.20–0.93	**<0.01**	−0.08 (11)	0.94	-		
CAM															
Insomnia severity (ISI)	3	3	294	79.2	0.35	9.62 (2)	<0.01	0.10	−1.53–1.73	0.82	**-**		-		
Sleep quality (PSQI)	14	17	1,173	94.6	1.63	294.22 (16)	<0.01	1.11	0.43–1.78	**<0.01**	2.52 (15)	**<0.05**	0.23	−0.58–1.05	0.56
Mindfulness-based															
Insomnia severity (ISI)	3	3	268	95.3	1.10	42.75 (2)	<0.01	0.26	−2.44–2.96	0.72	**-**		-		
Sleep quality (PSQI/MOS-S)	3	4	225	94.4	1.5	53.68 (3)	<0.01	0.06	−1.97–2.08	0.93	**-**		-		
Exercise-based															
Insomnia severity (ISI/AISI)	2	2	118	0.0	<0.01	0.02 (1)	0.89	−0.24	−0.57–0.095	0.07	**-**		-		
Sleep quality (PSQI)	3	4	217	91.3	1.17	34.30 (3)	<0.01	0.25	−1.56–2.05	0.69	**-**		-		
Herbal medicine															
Insomnia severity (ISI)	2	2	48	0.0	0.06	0.93 (1)	0.33	0.89	−2.85–4.64	0.20	**-**		-		
Relaxation															
Sleep quality (PSQI)	4	4	306	90.8	0.89	32.64 (3)	<0.01	1.18	−0.40–2.75	0.10	**-**		-		
BBT-I															
Insomnia severity (ISI)	2	2	152	85.0	0.55	6.67(1)	<0.01	0.95	−6.58–8.49	0.35	**-**		-		
Sleep quality (PSQI)	2	2	148	0.0	<0.01	0.44(1)	0.51	0.48	−1.01–1.96	0.15					

K=Number of studies, g = Hedge’s g; CBT-I=Cognitive Behavioural Therapy for Insomnia, CAM = Complementary and Alternative Medicine, BBT-I=Brief Behavioural Therapy for Insomnia; Bolded values indicate statistical significance.

#### Cognitive behavioural therapy for insomnia (CBT-I)

**Figure 2:**
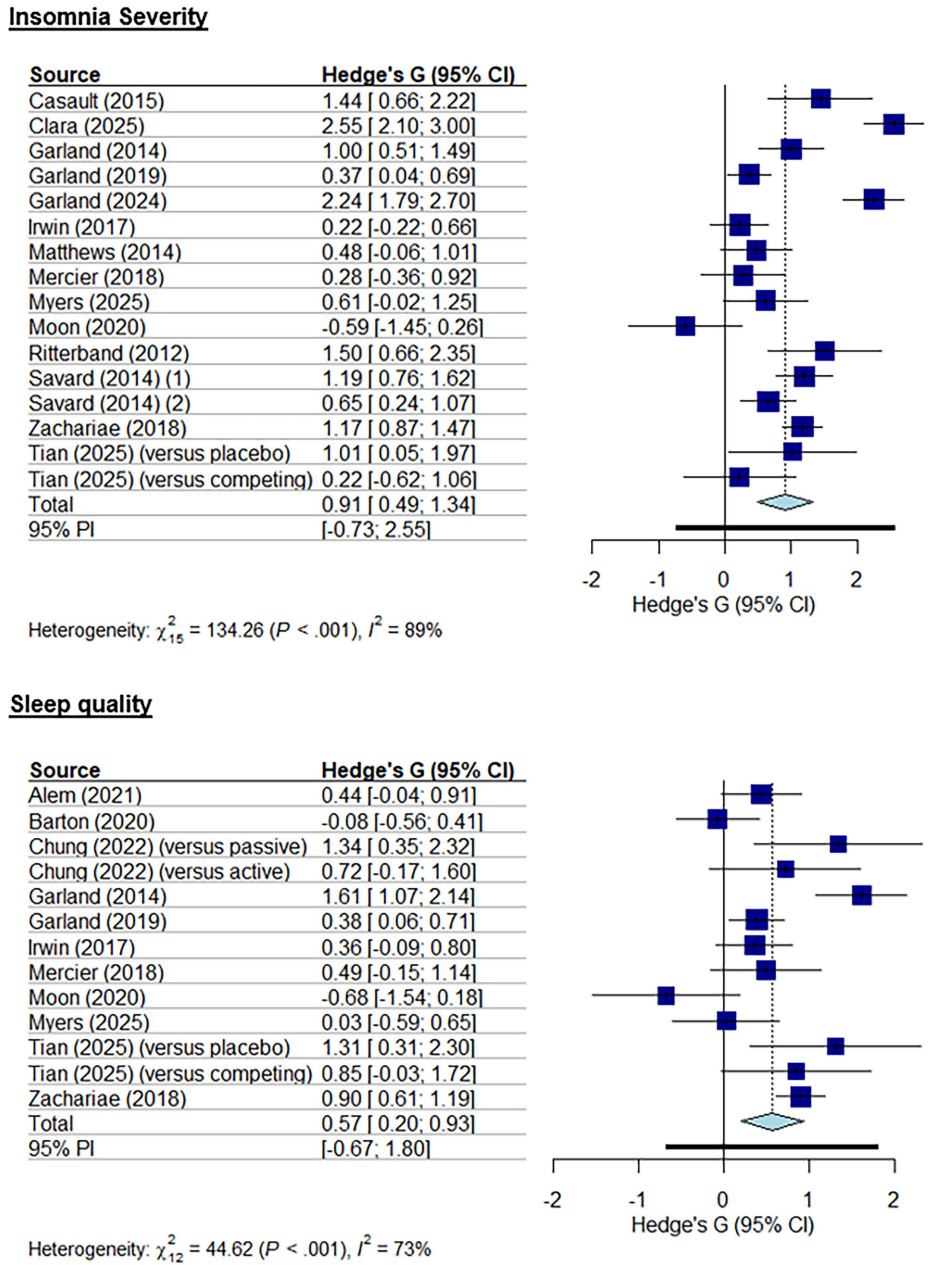
Forest plots for the effect of cognitive behavioural therapy for insomnia (CBT-I). Insomnia severity: Positive values (right side of the plot) indicate greater improvement with CBT-I compared to the comparator. For studies in which CBT-I served as the competing comparator (Garland et al., 2014; Garland et al., 2019; Irwin et al., 2017; Mercier et al., 2018; Moon et al., 2020; Tian et al., 2025), effect sizes were reversed to reflect the effect of CBT-I. Savard et al. (2014) (1): face-to-face CBT-I; Savard et al. (2014) (2): video-based CBT-I. The control group *N* in the study by Savard et al. (2014) was split to avoid a unit-of-analysis error. Tian et al. (2025) (versus competing): transcranial magnetic stimulation (rTMS); Tian et al. (2025) (versus placebo): sham rTMS. The shared intervention group *N* (CBT-I) in the study by Tian et al. (2025) was split to avoid a unit-of-analysis error. Sleep quality: Positive values (right side of the plot) indicate greater improvement with CBT-I compared to the comparator. For studies in which CBT-I served as the competing comparator (Garland et al., 2014; Garland et al., 2019; Irwin et al., 2017; Mercier et al., 2018; Moon et al., 2020; Tian et al., 2025), effect sizes were reversed to reflect the effect of CBT-I. Chung et al. (2022) (versus passive): waitlist control; Chung et al. (2022) (versus active): attentional control. Tian et al. (2025) (versus competing): transcranial magnetic stimulation (rTMS); Tian et al. (2025) (versus placebo): sham rTMS. The shared intervention group *N* (CBT-I) in both studies by Chung et al. (2022) and Tian et al. (2025) was split to avoid a unit-of-analysis error.

A statistically significant large ES of 0.91 (95% CI = 0.49–1.34, *p* < 0.01) for CBT-I was observed for managing insomnia severity as measured by the ISI or AISI at post-intervention, which was assessed in 16 comparisons across 14 independent randomized controlled trials (Table 2; Figure 2). Two comparisons were made in one trial by Savard et al. (2014) in which the effects of face-to-face CBT-I and video-based CBT-I were compared against a control group. Likewise, two comparisons were also made in another trial by Tian et al. (2025) in which the effects of CBT-I was compared against transcranial magnetic stimulation (rTMS) or sham rTMS. The shared control group *N* in the study by Savard et al. (2014), while the shared intervention group *N* (CBT-I) in the study by Tian et al. (2025) was split to avoid a unit-of-analysis error (Axon et al., 2023). A mean improvement of 8.04 points was found for CBT-I from baseline to post-intervention for the studies that had used ISI, with a possible range of scores of 0–28 (with Irwin et al. (2017) excluded, as they had used AISI). Egger’s test and the funnel plot did not detect any publication bias (*p =* 0.69) for the effects of CBTI on insomnia severity (Supplementary Figure 1). A sensitivity analysis was performed to include feasibility studies excluded during the selection process to examine whether they affected the ES or publication bias. Inclusion of two feasibility studies (Hall et al., 2022; Oswald et al., 2022) did not result in any major changes in ES (*g =* 0.93, 95% CI = 0.56–1.31, *p <* 0.01), which remained large and significant. Publication bias also remained nonsignificant (*p =* 0.72) (Supplementary Figure 8). The effectiveness of CBT-I on insomnia severity persisted at the short-term follow-up (≤3 months), as evidenced by seven trials that provided such data (*g =* 0.46, 95% CI = 0.05–0.87, *p <* 0.05). However, the effect of CBT-I did not remain significant in four studies with long-term follow-up data (6–12 months) (*g =* 0.34, 95% CI = −0.55–1.24 *p =* 0.31) (Table 3).

**Table 3:** Follow-up effects of cognitive behavioural therapy (CBT-I) and complementary and alternative medicine (CAM).

Intervention type	Short-term follow up							Long-term follow up						
Sleep outcome (measure)	K	Comparisons	*N*	I^2^	g	95% CI	*p*-value	K	Comparisons	*N*	I^2^	g	95% CI	*p*-value
CBT-I														
Insomnia severity (ISI/AISI)	7	7	605	74.3	0.46	0.05–0.87	**<0.05**	4	4	192	64.7	0.34	−0.55–1.24	0.31
Sleep quality (PSQI)	6	6	589	79.9	0.67	0.10–1.24	**<0.05**	2	2	106	62.6	−0.07	−4.46–4.32	0.87
CAM														
Sleep quality (PSQI)	5	6	411	37.2	0.07	−0.28–0.41	0.66	2	2	148	0	0.02	−0.27–0.31	0.50

K=Number of studies, g = Hedge’s g. Bolded values indicate statistical significance.

A statistically significant moderate ES of 0.57 (95% CI = 0.20–0.93, *p* < 0.01) for CBT-I was also observed for improving sleep quality as measured by the PSQI, which was assessed in 11 independent trials across 13 comparisons (Table 2; Figure 2). Two comparisons were made in two trials; in one trial, the effects of digital CBT-I were compared against an active control (attentional control) and passive control (waitlist control) (Chung et al., 2022), and in the other trial, the effect of CBT-I was compared against a competing intervention (rTMS) or a placebo (sham rTMS) (Tian et al., 2025). In both studies, the shared intervention group *N* (CBT-I) was split to avoid a unit-of-analysis error (Axon et al., 2023) (Table 2). A mean improvement of 3.88 points was found for the intervention from baseline to post-intervention, with a scoring range of 0–21 for the PSQI. Egger’s test and the funnel plot did not detect any publication bias for the effects of CBTI on sleep quality at post-intervention (Supplementary Figure 1). Similarly, the effectiveness of CBT-I in improving sleep quality persisted at the short-term follow-up, as evidenced by six trials that provided such data (*g =* 0.67, 95% CI = −0.10–1.24, *p < 0.05*), but not for the long-term follow-up (6–12 months), as evidenced by two trials (*g =* −0.07, 95% CI = −4.46–4.32, *p =* 0.87) (Table 3).

Given the considerable heterogeneity observed in the meta-analysis for CBT-I and insomnia severity (ISI) (*I*^2^ = 88.8%) and the availability of a sufficient number of studies (K ≥ 10), subgroup analyses were performed (Table 4). Participant characteristics, such as cancer type, age group, cancer treatment status and allowed use of sleep medication, had no significant influence on ES. Trials that had utilized passive comparators (e.g., routine care, waitlist control or no intervention) had a statistically significantly larger ES (*g =* 1.53; 95% CI = 0.91–2.15, *p <* 0.01) compared to active comparators (*g =* 0.53, 95% CI = −0.33–1.40, *p =* 0.08) and competing interventions (*g =* 0.31; 95% CI = −0.19–0.81, *p =* 0.17). Both remote and face-to-face interventions demonstrated significant effectiveness, with remote interventions showing a notably greater effect (*g =* 1.32, 95% CI = 0.64–1.99, *p <* 0.01) than face-to-face interventions (*g =* 0.52, 95% CI = 0.06–0.98, *p <* 0.05). Presence of staff engagement within remote interventions did not influence intervention effectiveness. Format (individual versus group) and risk of bias (low or some versus high) did not significantly influence the ES.

**Table 4:** Subgroup analyses of cognitive behavioural therapy (CBT-I) on insomnia severity.

	Subgroup analyses						
Covariates	K	Comparisons	g	I^2^	95% CI	Q (df)	*p*-value
Participant characteristics							
Age group^a^						1.03 (1)	0.31
Younger age (≤ 56.8 years)	6	8	1.12	87.00%	0.51–1.73		**<0.01**
Older age (> 56.8 years)	8	8	0.71	89.70%	0.51–1.73		0.05
Cancer type						0.52 (1)	0.47
Mixed	9	10	1.03	91.90%	0.33–1.72		**<0.01**
Breast	4	5	0.76	77.00%	0.23–1.30		**<0.05**
Prostate	1	1	Insufficient studies				
Cancer treatment status						2.07 (1)	0.15
Completed primary treatment	12	14	0.86	90.00%	0.29–1.21		**<0.01**
Not completed primary treatment	2	2	1.21	0.00%	−0.03–2.44		0.05
Sleep medication						1.00 (1)	0.32
Not allowed	3	4	0.52	77.50%	−0.92–1.96		0.33
Allowed	11	12	1.02	90.60%	0.54–1.51		**<0.01**
Intervention characteristics							
Delivery						5.34 (1)	**0.02**
Face-to-face	7	8	0.52	70.40%	0.06–0.98		**<0.05**
Remote	8	8	1.32	90.00%	0.64–1.99		**<0.01**
Remote engagement						0.05 (1)	0.82
With staff engagement	6	6	1.31	92.60%	0.32–2.30		**<0.05**
No staff engagement	2	2	1.22	0.00%	−0.32–2.75		0.06
Format						0.63 (1)	0.43
Individual	12	14	0.96	89.40%	0.48–1.44		**<0.01**
Group	2	2	0.60	81.20%	−4.34–5.55		0.37
Study characteristics							
Comparator						16.38 (2)	**<0.01**
Passive	6	7	1.53	88.70%	0.91–2.15		**<0.01**
Active	2	2	0.53	0.00%	−0.33–1.40		0.08
Competing	6	6	0.31	57.60%	−0.19–0.81		0.17
Placebo	1	1	Insufficient studies				
Risk of bias						3.63 (1)	0.06
Low or some concerns	5	6	0.52	33.00%	0.13–0.90		**<0.05**
High risk	9	10	1.13	91.60%	0.48–1.78		**<0.01**

a 56.8 is median of sample age of the studies in the current meta-analysis.

K=Number of studies, g = Hedge’s g.

Bolded values indicate statistical significance.

Subgroup analyses were also performed for the meta-analysis of CBT-I and sleep quality (PSQI) given its considerable heterogeneity (*I*^2^ = 73.1%). Participant characteristics, such as cancer type, age group, cancer treatment status, use of sleep medication, as well as intervention characteristics, including delivery, individual versus group format, comparator type, and risk of bias had no significant influence on ES (Table 5).

**Table 5:** Subgroup analyses of cognitive behavioural therapy (CBT-I) on sleep quality.

	Subgroup analyses						
Covariates	K	Comparisons	g	I^2^	95% CI	Q (df)	*p*-value
Participant characteristics							
Age group^a^						1.16 (1)	0.28
Younger age (57.1 ≤ years)	5	7	0.79	0.0%	0.49–1.08		**<0.01**
Older age (> 57.1 years)	5	5	0.38	85.0%	−0.63–1.38		0.36
Cancer type						0.02 (1)	0.89
Mixed	8	10	0.61	74.8%	0.13–1.09		**<0.05**
Breast	2	2	0.66	75.1%	−2.76–4.07		0.25
Prostate	1	1	Insufficient studies				
Cancer treatment status						1.31 (1)	0.25
Completed primary treatment	8	9	0.46	77.0%	−0.06–0.99		0.08
Not completed primary treatment	3	4	0.78	22.6%	0.27–1.30		**<0.05**
Sleep medication						0.05 (1)	0.83
Not allowed	4	6	0.53	75.1%	−0.31–1.36		0.17
Allowed	7	7	0.61	71.8%	0.15–1.07		**<0.05**
Intervention characteristics							
Delivery						0.22 (1)	0.64
Face-to-face	7	8	0.51	78.8%	−0.08–1.10		0.08
Remote^b^	4	5	0.66	50.7%	0.10–1.23		**<0.05**
Format						0.45 (1)	0.50
Individual	7	10	0.49	68.6%	0.06–0.92		**<0.05**
Group	4	3	0.79	86.2%	−0.94–2.52		0.19
Study characteristics							
Comparator						4.55 (2)	0.10
Passive	3	3	0.80	47.9%	−0.16–1.76		0.07
Active	3	3	0.13	17.3%	−0.82–1.08		0.61
Competing	6	6	0.53	80.0%	−0.23–1.28		0.13
Placebo	1	1	Insufficient studies				
Risk of bias						2.55 (1)	0.11
Low or some concerns	4	5	0.89	72.3%	0.19–1.59		**<0.05**
High risk	7	8	0.37	72.7%	−0.11–0.85		0.11

a 57.1 is median of sample age of the studies in the current meta-analysis; Barton et al. (2020) omitted due to missing covariate data.

b No further subgroup analysis was conducted for level of staff engagement within remote interventions as only one study (Zachariae et al., 2018) had no staff engagement.

K=Number of studies, g = Hedge’s g.

Post-hoc analyses were conducted to examine remission rates of insomnia for CBT-I. Only studies that reported remission rates in accordance with their initial screening criteria were included in the analysis; for example, if the study enrolled participants with an ISI score ≥8, remission was defined as scoring below this threshold. Three such studies met this criterion; whereby they provided remission rates defined as the proportion of participants scoring below the clinical cut off of ISI score of 8 at post-intervention (Casault et al., 2015; Mercier et al., 2018; Savard et al., 2014). Individuals receiving CBT-I were nearly 4 times more likely to achieve remission by scoring below the ISI clinical cut-off of 8 than their comparators (OR = 3.94, 95% CI = 1.44–10.74, *p <* 0.01), with remission rates averaging at 57.3% for CBT-I and 27.4% for their comparators. Remission rates based on the PSQI were not examined due to insufficient reporting across the studies (K < 2).

#### Complementary and alternative medicine (CAM)

**Figure 3:**
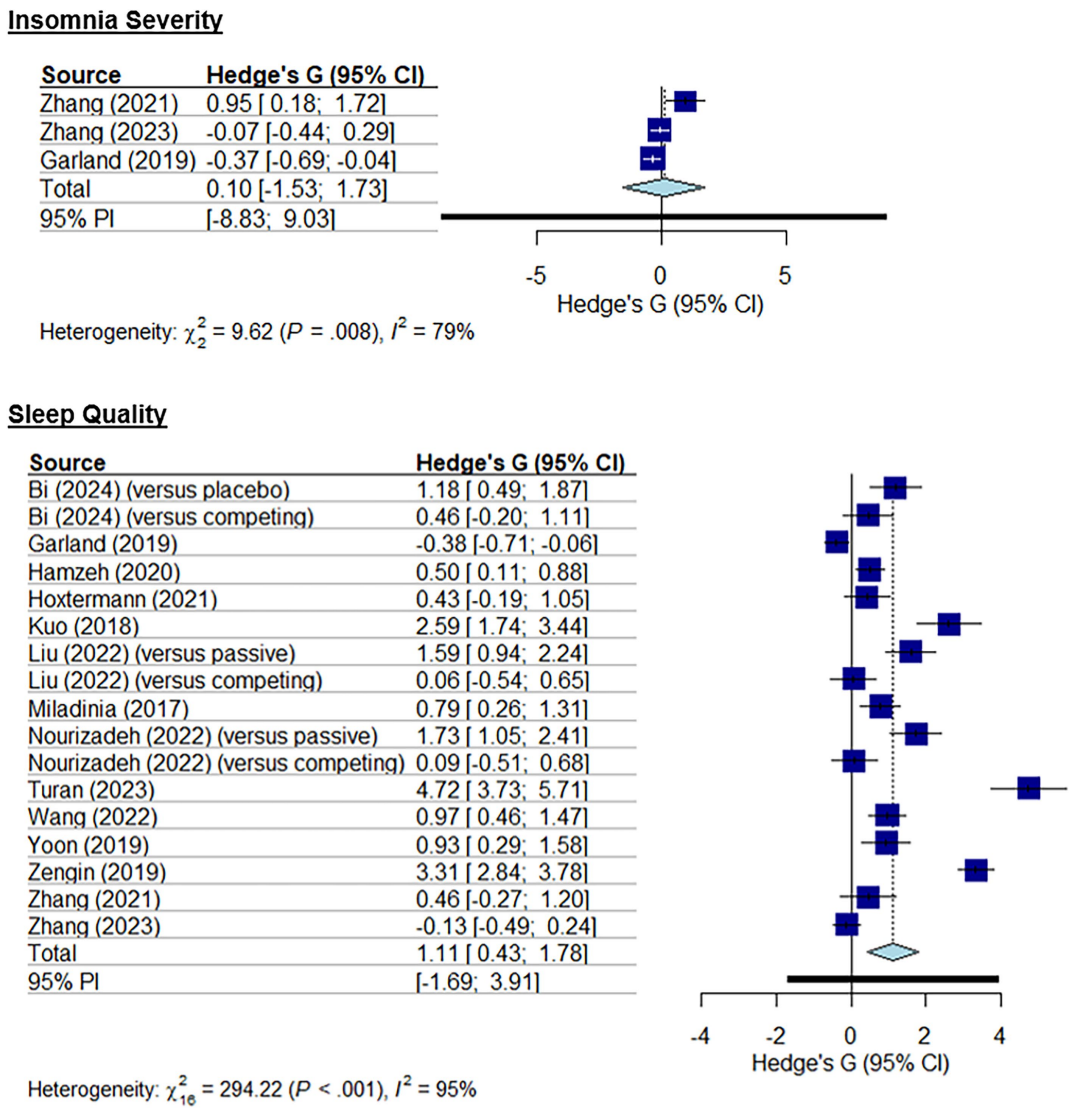
Forest plots for the effect of complementary and alternative medicine (CAM) Sleep quality. Positive values (right side of the plot) indicate greater improvement with CAM compared to the comparator. Bi et al. (2024) (versus placebo): placebo auricular acupressure; Bi et al. (2024) (versus competing): positive psychological intervention. Liu et al. (2022) (versus passive): routine care; Liu et al. (2022) (versus competing): mindfulness-based stress reduction. Nourizadeh et al. (2022) (versus passive): routine care; Nourizadeh et al. (2022) (versus competing): aerobic exercise training. The shared intervention group *N* (acupressure) was split to avoid a unit-of-analysis error for Bi et al. (2024), Liu et al. (2022), and Nourizadeh et al. (2022).

A nonsignificant, small ES of 0.10 (95% CI = −1.53–1.73, *p =* 0.82) for CAM was observed for managing insomnia severity (ISI), which was assessed in three independent trials (Table 2; Figure 3). A sensitivity analysis was performed to include two feasibility studies excluded during the selection process to examine whether they affected the ES, which examined the effect of electroacupuncture (Lee et al., 2022) and self-acupressure (Huong et al., 2022) respectively. The inclusion of these two studies also resulted in a small and non-significant effect (*g =* 0.36, 95% CI = −0.23–0.95, *p =* 0.18) (Supplementary Figure 9).

In contrast, a statistically significant large ES of 1.11 (95% CI = 0.43–1.78, *p* < 0.01) for CAM for improving sleep quality was observed, as measured by the PSQI at post-intervention, which was assessed in 17 comparisons across 14 independent randomized controlled trials (Table 2; Figure 3). As two trials (Liu et al., 2022; Nourizadeh et al., 2022) simultaneously compared the effect of acupressure against both a passive control and a competing intervention, and a third trial compared the effect of acupressure against both a placebo (sham control) and a competing intervention (Bi et al., 2024), the shared intervention group *N* (acupressure) of these trials was split to avoid a unit-of-analysis error (Axon et al., 2023). Thus, each trial contributed two separate comparisons: the first trial included acupressure versus passive control (routine care) and acupressure versus a competing intervention (mindfulness-based stress reduction) (Liu et al., 2022), the second trial included self-acupressure versus passive control (routine care) and self-acupressure versus a competing intervention (aerobic exercise training) (Nourizadeh et al., 2022), and the third trial included acupressure versus placebo (sham control) and acupressure versus a competing intervention (positive psychological intervention) (Bi et al., 2024). A mean improvement of 5.19 points on the PSQI was found for the intervention from baseline to post-intervention for CAM.

Egger’s test and the funnel plot indicated the presence of publication bias for CAM on improving sleep quality (PSQI) (*p* < 0.05) (Supplementary Figure 2) and the Duval and Tweedie trim-and-fill method was used to produce an adjusted ES (Table 2). Seven outliers were identified, and the analysis reported an adjusted non-significant small ES of 0.23 (95% CI = −0.58–1.05, *p =* 0.56) for CAM in improving sleep quality (PSQI). A sensitivity analysis was performed to include two feasibility studies excluded during the selection process to examine whether they affected the ES or publication bias. The two feasibility studies examined the effects of bright light therapy (Fox et al., 2021) and electroacupuncture (Lee et al., 2022) respectively. There were no major changes to the ES (*g =* 1.13, 95% CI = 0.53–1.72, *p <* 0.01), which remained large and significant. Publication bias (*p <* 0.05) also remained significant (Supplementary Figure 10), and the trim-and-fill analysis similarly produced a non-significant small ES of 0.20 (95% CI = −0.52–0.92, *p =* 0.58). The effectiveness of CAM did not persist at short-term follow-up, as evidenced by four trials (*g =* 0.07, 95% CI = −0.28–0.41, *p* = 0.66), nor long-term follow-up, as evidenced by two trials (*g =* 0.02, 95% CI = −0.27–0.31, *p* = 0.50) (Table 3).

Subgroup analyses were conducted as considerable heterogeneity was reported for CAM in improving sleep quality (I^2^ = 94.6%) and availability of a sufficient number of studies (Table 6). Given that the majority of trials under CAM examined the effect of acupressure or acupuncture, a subgroup analysis was performed separately for acupuncture or acupressure versus other CAM interventions, including essential oil (Hamzeh et al., 2020), slow-stroke back massage (Miladinia et al., 2017), and reflexology (Zengin and Aylaz, 2019). There was no significant difference in ES between acupuncture or acupressure versus other CAM interventions. Participant characteristics, such as age, cancer type and allowed use of sleep medication did not significantly influence the ES. In contrast, undergoing primary cancer treatment had a significantly larger ES (*g =* 1.64; 95% CI = 0.61–2.66, *p <* 0.01) than completed primary treatment (*g =* 0.44; 95% CI = −1.00–1.87, *p =* 0.40) or not completed primary treatment (*g =* 0.08, 95% CI = −3.49–3.65, *p =* 0.83). Regarding intervention characteristics, studies that had passive control conditions had a statistically significant larger ES (*g =* 2.08, 95% CI = 0.40–3.76, *p* < 0.05) compared to active controls (*g =* 1.30; 95% CI = −1.46–4.05, *p =* 0.18), placebo (*g =* 0.57; 95% CI = −0.35–1.48, *p =* 0.14) and competing interventions (*g =* −0.02; 95% CI = −0.59–0.55, *p =* 0.92). Furthermore, studies that were identified as high risk of bias were significantly associated with a higher ES (*g =* 1.38; 95% CI = 0.44–2.32, *p <* 0.01) compared to studies with low or some risk of bias (*g =* 0.44; 95% CI = −0.13–1.01, *p =* 0.10). Post-hoc analyses on remission rates of CAM were not examined due to insufficient reporting across studies (K < 2).

**Table 6:** Subgroup analyses of complementary and alternative medicine (CAM) on sleep quality.

	Subgroup analyses						
Covariates	K	Comparisons	g	I^2^	95% CI	Q (df)	*p* value
Participant characteristics							
Age group^a^						0.78 (1)	0.38
Younger age (≤ 51.2 yrs)	6	8	0.70	67.0%	0.23–1.16		**<0.01**
Older age (> 51.2 years)	8	8	1.24	95.2%	−0.15–2.63		0.07
Cancer type						3.17 (2)	0.20
Mixed	4	5	1.01	97.6%	−0.74–2.76		0.18
Breast	7	9	0.66	81.7%	0.15–1.18		**<0.05**
Others	3	3	2.67	96.1%	−2.22–7.56		0.14
Cancer treatment^b^						8.55 (2)	**<0.05**
Completed primary treatment	3	4	0.44	90.6%	−1.00–1.87		0.40
Undergoing primary treatment	8	10	1.64	94.1%	0.61–2.66		**<0.01**
Not completed primary treatment	2	2	0.08	49.3%	−3.49–3.65		0.83
Sleep medication^c^						0.13 (1)	0.72
Not allowed	7	10	1.03	93.2%	0.31–1.75		**<0.01**
Allowed	6	6	1.33	96.2%	−0.69–3.36		0.15
Intervention characteristics							
Subtype						0.29 (1)	0.59
Acupressure or acupuncture	11	14	1.01	92.3%	0.26–1.77		**<0.05**
Essential oil, massage, reflexology	3	3	1.53	97.8%	−2.32–5.38		0.23
Study characteristics							
Comparator						13.74 (3)	**<0.01**
Passive	6	6	2.08	94.9%	0.40–3.76		**<0.05**
Active	3	3	1.30	88.0%	−1.46–4.05		0.18
Placebo	4	4	0.57	81.0%	−0.35–1.48		0.14
Competing	4	4	−0.02	52.8%	−0.59–0.55		0.92
Risk of bias						3.94 (1)	<0.05
Low or some concerns	4	5	0.44	68.8%	−0.13–1.01		0.10
High risk	10	12	1.38	95.8%	0.44–2.32		**<0.01**

a 51.2 is median of sample age in current meta-analysis; Zengin and Aylaz (2019) excluded due to missing covariate data.

b Hamzeh et al. (2020) omitted due to missing covariate data.

c Miladinia et al. (2017) omitted due to missing covariate data.

K=Number of studies, g = Hedge’s g.

#### Other interventions

Three independent trials examined the effect of mindfulness on managing insomnia severity (ISI) and a non-significant effect was found (*g =* 0.26, 95% CI = −2.44–2.96, *p =* 0.72). Similarly, mindfulness did not yield significant effects on improving sleep quality (PSQI/MOS-S) (*g =* 0.06, 95% CI = −1.97–2.08, *p =* 0.93) in four comparisons across the three independent trials (Table 2; Supplementary Figure 3). The effect of exercise-based interventions on insomnia severity (ISI/AISI) was only assessed in two independent trials, whereby both trials used competing interventions of CBT-I, yielding a non-significant ES of −0.24 (95% CI = −0.57–0.09, *p =* 0.07). The effects of exercise on sleep quality (PSQI) were also examined in four comparisons in three independent trials and similarly yielded a non-significant effect of 0.25 (95% CI = −1.56–2.05, *p =* 0.69) (Table 2; Supplementary Figure 4). The effects of herbal medicine on insomnia severity (ISI) were examined in two independent trials, which yielded a non-significant large ES of 0.89 (95% CI = −2.85–4.64, *p =* 0.20) (Table 2; Supplementary Figure 5). The effect of relaxation interventions was assessed in four independent trials on improving sleep quality (PSQI). Similarly, a large yet nonsignificant effect was found (*g =* 1.18, 95% CI = −0.40–2.75, *p =* 0.10) (Table 2; Supplementary Figure 6). Lastly, brief behavioural therapy for insomnia (BBT-I) on managing insomnia severity (ISI) and improving sleep quality (PSQI) was assessed in two independent trials. BBT-I had a large yet nonsignificant effect on reducing insomnia severity (*g =* 0.95, 95% CI = −6.58–8.49, *p =* 0.35) (Table 2; Supplementary Figure 7). Inclusion of an additional feasibility study examining BBT-I (Palesh et al., 2018) did not result in major change in ES (*g =* 0.82, 95% CI = −0.69–2.32, *p =* 0.14) (Supplementary Figure 11). BBT-I also demonstrated a moderate and nonsignificant effect on improving sleep quality (*g =* 0.48, 95% CI = −1.01–1.96, *p =* 0.15) (Table 2; Supplementary Figure 7). No subgroup analyses were conducted for the above-mentioned intervention types as there were insufficient trials available (K < 10).

As only one study examined the effect of behavioural activation (Li et al., 2024), positive psychological intervention (Bi et al., 2024), transcranial magnetic stimulation (Tian et al., 2025) and pharmacological medication (melatonin versus zolpidem) (Shahrokhi et al., 2021), meta-analyses were not feasible. Instead, the intervention effects were first summarised qualitatively as according to the results of original study, followed by independent calculation of individual effect sizes using Hedges’ g to facilitate a more meaningful relative comparison.

Behavioural activation was reported to be significantly more effective than usual care in improving sleep quality as measured by the PSQI, consistent with our ES calculations, whereby a moderate positive effect was found (*g =* 0.60, 95% CI = 0.20–1.00).

Positive psychological intervention (PPI) was significantly more effective than sham auricular acupressure (AA) for improving sleep quality (PSQI), but less effective than active AA. This aligns with our ES calculations: when comparing PPI against sham AA, a large, positive, significant effect was found (*g =* 0.71, 95% CI = 0.71–1.26). When PPI was compared against AA, a negative, nonsignificant effect was found (*g =* −0.46, 95% CI = −0.10–0.07).

Transcranial magnetic stimulation (rTMS) showed significant improvements in reducing insomnia severity (ISI) and sleep quality (PSQI) when compared against sham rTMS but not CBT-I. Our ES calculations support this, finding that when compared against sham rTMS, a large, positive, significant effect was found for insomnia severity (*g =* 0.88, 95% CI = 0.13–1.62) and a moderate but nonsignificant effect for improving sleep quality (*g =* 0.62, 95% CI = −0.10–1.35). When comparing rTMS against CBT-I, a negative, nonsignificant effect was found for insomnia severity (*g =* −0.22, 95% CI = −0.91–0.48), but a negative significant effect was found for sleep quality (*g =* −0.86, 95% CI = −1.58–−0.13).

Both zolpidem and melatonin were observed to have significant impacts on sleep quality at post intervention using the PSQI however there was no significant difference in the effectiveness between zolpidem and melatonin at 8 weeks follow-up (4 weeks post-intervention). Consistent with our ES calculations, a large effect favouring zolpidem against melatonin was found (*g =* 1.18; 95% CI = 0.23–1.63) at post intervention, however the effect was diminished at follow up (4 weeks post-intervention), with no significant difference between the two groups (*g =* −0.90; 95% CI = −0.50–0.32). Discrepancies in statistical significance may be attributable to our use of Hedges’ g, which adjusts for small sample sizes and relies on unadjusted post-intervention data.

#### Risk of bias

**Figure 4:**
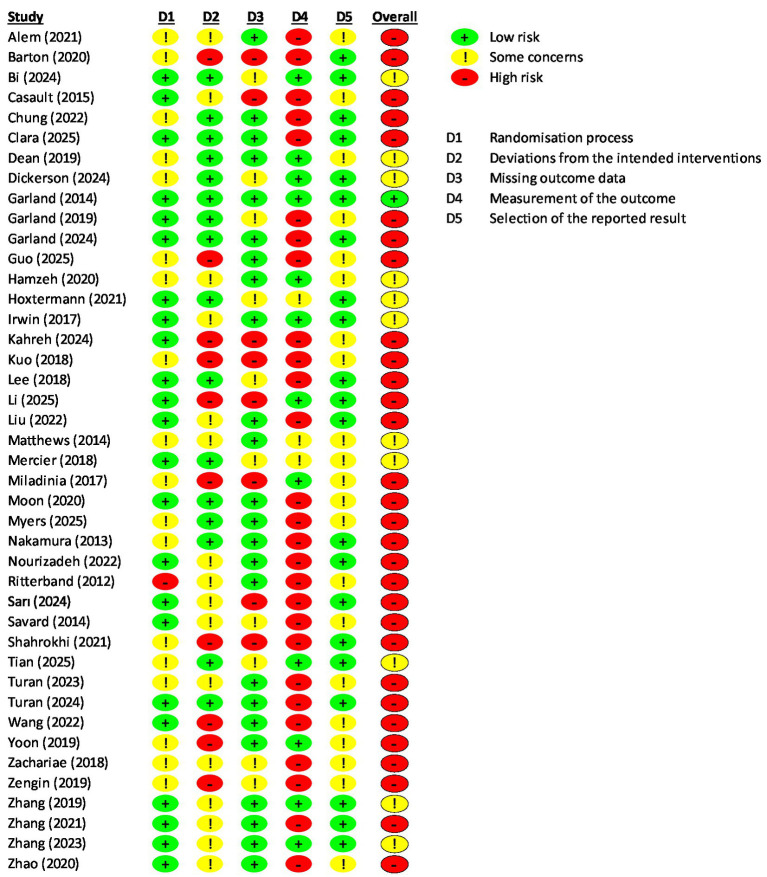
Risk of bias assessments (version 2).

The risk of bias assessment is shown in Figure 4. Thirty studies (71.4%) were characterized as having an overall high risk of bias, 11 studies (26.2%) were characterized as having some concerns and one study (2.4%) had a low risk of bias. Most studies that had high risk of bias were influenced by biases related to the measurement of the outcome, due to studies using a passive control condition (e.g., waitlist control, no intervention). Furthermore, some studies showed bias in deviations from the intended interventions due to a lack of blinding. Biases in missing outcome data were also reported when the dropout rate between the intervention and control groups was notably imbalanced.

#### Certainty of evidence

Supplementary Table 2 shows a summary of the overall certainty of evidence. Certainty in the evidence was rated “very low” for all outcomes per intervention type and per follow-up period. This was due to most included studies being characterized as high risk of bias, and considerable heterogeneity (I^2^) reported from the meta-analyses, which remained even after subgroup analyses, particularly for CBT-I on sleep quality. Further, given that the current review examined different types of cancer types, sleep disturbance requirements (e.g., diagnostic criteria for insomnia, PSQI >5), as well as comparisons groups (e.g., active control, passive control, competing control), the level of certainty was downgraded to “very serious” regarding inconsistency. Lastly, as some meta-analyses (e.g., exercise, herbal medicine, mindfulness-based intervention) showed wide 95% CIs demonstrating little to no effect of the studied outcomes and had less than 5 studies included, the level of certainty was downgraded to “very serious” regarding imprecision.

## Discussion

This systematic review and meta-analysis consolidated up-to-date evidence on the effectiveness of existing interventions targeted at managing clinically relevant levels of sleep disturbance or insomnia severity in patients with cancer and survivors at post-intervention in the past decade. This included a variety of interventions, consisting of cognitive behavioural therapy (CBT-I), complementary and alternative medicine (CAM), mindfulness-based interventions, exercise, herbal medicine, brief-behavioural therapy for insomnia (BBT-I), relaxation and pharmacotherapy, which were primarily examined using PSQI and ISI.

The present meta-analysis found a moderate effect size for CBT-I on sleep quality (*g =* 0.57) and a larger effect size for insomnia severity (*g =* 0.91). These effect sizes are consistent with previous meta-analyses conducted on CBT-I in cancer at post-intervention, particularly with a recent meta-analysis conducted by Nissen et al. (2024) who reported effect sizes of 0.86 and 0.52 for insomnia severity and sleep quality, respectively. Squires et al. (2022) (*g* = 0.78), Johnson et al. (2016) (*d* = 0.77), Ma et al. (2021) (*g* = −0.78) similarly reproduced similar effect sizes in reducing insomnia severity. The mean improvement from baseline to post-intervention of 8.04 on the ISI and 3.88 on the PSQI is within the threshold(s) for clinically meaningful change of 6–8.4 points on the ISI (Morin et al., 2011; Lenderking et al., 2024) and 3 points on the PSQI (Buysse et al., 1989) respectively. The comparatively stronger effect observed of CBT-I on ISI relative to PSQI may be attributed to the differences in the psychometric properties of these instruments. Both the ISI and PSQI measure sleep latency, sleep maintenance, sleep satisfaction, and daytime impairment; however, the ISI specifically addresses the fundamental cognitive and behavioural symptoms of insomnia, including perceived distress or worry about sleep, which the CBT-I is designed to target (Altena et al., 2023). Meanwhile, the PSQI captures broader dimensions of sleep, including sleep disruptions such as pain, breathing difficulties, and nocturia. As these latter components are influenced by factors that CBT-I is not designed to directly target, PSQI scores may remain elevated, even as insomnia-specific symptoms improve. Thus, the effect of CBT-I in reducing ISI may be greater than that of the PSQI.

Subgroup analyses of CBT-I on managing insomnia severity revealed no significant difference in the effectiveness of CBT-I based on format (group vs. individual) but found significant difference between different delivery methods (in-person vs. remote) of CBT-I, whereby remote interventions had a greater effect than face-to-face interventions. However, this difference is likely attributable to the types of comparators used, as many face-to-face interventions were compared against competing interventions whilst remote interventions were more often evaluated against passive controls. Despite this difference, both face-to-face and remote interventions were found to be significantly effective, indicating that CBT-I can be successfully delivered across different modalities. There was also no significant difference in ES between those with or without staff engagement (e.g., calls from researchers to answer questions or to maintain adherence and therapist-guided intervention over video call) amongst remote CBT-I interventions. These findings are consistent with those of previous meta-analyses that examined the effect of CBT-I on insomnia in cancer populations (Ma et al., 2021; Nissen et al., 2024). Several studies included in the present meta-analysis used self-help or self-administered versions of the CBT-I (Casault et al., 2015; Mercier et al., 2018; Savard et al., 2014), which included videos and printed materials. These findings indicate that CBT-I allows for a high degree of flexibility in implementation, and that remote-based CBT-I is a practical option for improving access to care.

Furthermore, CBT-I was found to be more effective when compared against passive controls in managing insomnia severity; however, a non-significant effect was found when compared with active and competing controls. This suggests that while CBT-I is effective, its superiority may be context-dependent. It is plausible that mindfulness or exercise-based interventions, may provide some benefits for managing insomnia; however, they may not be as effective as CBT-I. This is particularly evident in our meta-analysis assessing the effect of exercise on insomnia severity; exercise was found to have an overall negative effect compared with CBT-I in two trials (Irwin et al., 2017; Mercier et al., 2018), which was borderline significant (*g =* −0.24, *p =* 0.07).

Additionally, subgroup analyses did not reveal a significant difference in effect size based on cancer treatment status, suggesting that CBT-I is suitable for both cancer patients undergoing treatment or for cancer survivors at the post-treatment phase. Lastly, CBT-I did not show differences in effectiveness in relation to cancer type, age group, nor allowed use of sleep medication, suggesting that the benefits of these interventions are applicable across diverse cancer populations for managing insomnia.

Subgroup analyses of CBT-I on sleep quality (PSQI) did not reveal any significant effect modifiers. A possible explanation for this discrepancy may again be attributable to the differences in psychometric properties between the ISI and the PSQI. Chen et al. (2017) found that the ISI had stronger measurement invariance against CBT-I, whereas the PSQI exhibited poorer model fit and greater non-invariance. As CBT-I emphasises on cognitive restructuring and targets dysfunctional beliefs and attitudes related to sleep (Altena et al., 2023), the ISI may be more sensitive to treatment effects as it directly focuses on the respondents’ subjective perceptions about insomnia symptoms rather than general sleep quality. As such, the absence of significant subgroup effects may reflect limitations in the sensitivity of the PSQI to detect treatment effects, rather than an absence of underlying differences.

While the effectiveness of CBT-I for improving sleep quality and insomnia severity was shown to persist at short-term follow-up post-intervention (≤3 months), it was not found at long-term follow-up (> 3 months), suggesting that patients may benefit from the ongoing reinforcement of CBT-I techniques. Booster sessions may help to maintain the gains achieved during the initial therapy. Future studies may consider developing adaptive stepped care trial designs to provide a low-intensity, remote-based treatment, and depending on patient response, a higher-intensity, in-person intervention may be provided if necessary. A recent non-inferiority randomized controlled trial found that cancer patients with mild-to-moderate insomnia who initially received a web-based CBT-I intervention, followed by face-to-face CBT-I if needed, demonstrated significant improvement in sleep outcomes that were not significantly inferior compared with standard therapist-led CBT-I (Savard et al., 2021). Sequential multiple assignment randomized trials (SMART) may further optimize treatment strategies, so patients receive the most efficient interventions dependent on their needs. For example, previous SMART trials that have been implemented in cancer survivors for symptom management have demonstrated significant improvement in psychological distress (Badger et al., 2024; Sikorskii et al., 2023), suggesting the potential of implementing CBT-I as a SMART trial to manage sleep disturbances.

A large effect size was found for the effect of CAM on improving sleep quality as measured by the PSQI (*g =* 1.11), which was primarily characterized by acupuncture and acupressure therapies. The mean improvement from baseline to post-intervention of 5.19 points on the PSQI also signifies clinically meaningful changes in reducing sleep disturbances. However, CAM produced a non-significant improvement in insomnia severity as measured by the ISI. Unlike CBT-I, the effects of CAM on sleep quality did not persist at either short-term or long-term follow-up. These findings are consistent with those of previous meta-analyses (Guo et al., 2024; Weng et al., 2024; Yu et al., 2022), which mutually found that acupuncture was only effective in reducing PSQI scores but not ISI scores. These findings may be attributed to the differences in the working mechanisms between CAM and CBT-I. CAM may provide temporary relief by targeting acupoints in relieving physical side effects such as fatigue, pain, nausea or vomiting, factors known to directly impact sleep disturbance (He et al., 2020). However, CAM may not provide sustainable effects because it does not target perpetuating factors, such as cognitive or behavioural factors (e.g., pre-sleep arousal and sleep-related dysfunctional beliefs) that contribute to chronic insomnia (Altena et al., 2023). Additionally, under the assumption that CAM relieves physical side effects, improvements in the PSQI may be more pronounced than in the ISI as the PSQI assesses physical symptoms, such as pain, while the ISI does not. Furthermore, one study (Zhang et al., 2023) included in our meta-analysis found no difference in effect between sham and acupuncture, suggesting that acupuncture in nonspecific acupoints (acupoints irrelevant to the targeted condition) may still bring benefits to improving sleep quality (Linde et al., 2010). This aligns with a recent meta-analysis, which found that sham acupuncture was ranked higher in effectiveness than manual acupuncture or electroacupuncture for treating sleep disturbances in cancer patients (Cheung et al., 2022).

Subgroup analyses were also conducted to assess the performance of CAM when compared against different comparators given the substantial heterogeneity observed. CAM demonstrated notable effectiveness in improving sleep quality when faced with passive control conditions; however, its effectiveness was largely reduced when faced with active, placebo (sham), and competing interventions (e.g., CBT-I, mindfulness, and aerobic exercise). While a similar trend was observed for CBT-I, the pooled effect size of CAM versus competing interventions was negative (*g =* −0.02), as opposed to CBT-I, which maintained a positive effect size for insomnia severity (*g =* 0.31) and sleep quality (*g =* 0.53) respectively, although it was not significant. CAM may have beneficial effects, but its relative effectiveness may diminish, particularly when compared with other established interventions. Nevertheless, these results provide preliminary evidence of the potential benefits of CAM. Additionally, undergoing primary treatment was found to be significantly more effective for CAM; suggesting that CAM may be effective in addressing immediate cancer treatment-related side effects.

Other intervention types, including mindfulness, exercise-based interventions, herbal medicine, brief behavioural therapy for insomnia (BBT-I) and relaxation did not show significant effects on managing insomnia severity or improving sleep quality. Among these, relaxation interventions and Brief Behavioural Therapy for Insomnia (BBT-I), a less intensive variant of Cognitive Behavioural Therapy for Insomnia (CBT-I), yielded a large, yet statistically nonsignificant effect size in improving sleep quality (*g =* 1.18) and insomnia severity (*g =* 0.95), respectively. However, it should be noted that these findings should be interpreted with caution as there were a limited number of studies available, particularly for exercise-based interventions, herbal medicine and BBT-I (K = 2). Further randomized controlled trials are warranted to evaluate its effects. Importantly, future research should prioritize direct comparisons against more established interventions, such as CBT-I. For example, existing studies examining BBT-I have largely been limited to comparisons with active controls such as healthy eating interventions.

As only one study has examined the effect of pharmacotherapy (melatonin versus zolpidem), transcranial magnetic stimulation, behavioural activation and positive psychological intervention respectively, its overall effectiveness remains inconclusive. The scarcity of randomised controlled trials investigating the effect of pharmacological treatments for insomnia in cancer populations is unsurprising. A recent review by Pinucci et al. (2023) identified 15 studies that addressed pharmacological treatment for insomnia in cancer patients, encompassing randomised controlled trials, systematic reviews, and meta-analyses; however, only four of these were randomised controlled trials, which did not target clinically relevant sleep disturbance or insomnia. Notably, the studies included in the single meta-analysis within that review did not directly evaluate sleep outcomes. Instead, it focused on the use of olanzapine for chemotherapy-induced nausea. Thus, our current review underscores the limited nature of the current evidence on the efficacy of pharmacological treatments for clinically relevant sleep disturbances or insomnia in cancer populations. Furthermore, while a significant number of randomized controlled trials were conducted to examine the effects of different interventions beyond CBT-I or CAM (e.g., melatonin, physical activity), as evidenced by previous meta-analyses (Fan et al., 2022; Nissen et al., 2024; Luo et al., 2024; Yang et al., 2021), many did not require a clinical or diagnostic requirement for insomnia; as a result, they were not included in the present meta-analysis. Future interventional studies should focus on expanding the evidence base by conducting more randomized controlled trials that specifically address patients with insomnia or clinical levels of sleep disturbance to better determine the effectiveness of various interventions with greater precision.

Additionally, more than half of the included studies permitted participants to use sleep medications during the trial period. Amongst the studies that permitted sleep medication, interventions included CBT-I, CAM, mindfulness, exercise, herbal medicine as well as relaxation. Although the subgroup analyses did not reveal any significant differences in effect sizes between studies that had permitted sleep medication compared to those that did not for both CBT-I and CAM, studies that allowed concurrent use of sleep medications demonstrated larger effects. This suggests that in many cases, interventions were intended to be implemented as adjuncts to existing pharmacotherapy regiments, and the observed effects of the subgroup analysis reflects the complementary effects of CBT-I and CAM when used alongside sleep medication. Future randomised controlled trials should attempt to further delineate the effectiveness of non-pharmacological interventions when used as adjuncts to, or alternatives for sleep medication.

A major strength of the present meta-analysis was its stringent inclusion criteria for presence of clinical sleep disturbance or insomnia, which ensured that only studies with clinically relevant populations were included in the analysis. This meticulous selection process provided more meaningful insights into whether the included interventions can effectively reduce clinical sleep disturbance and insomnia severity, rather than just improving sleep quality in cancer patients and survivors. Further, the present meta-analysis is the first to examine the effectiveness of CAM, as previous reviews have focused on specific subtypes of treatment, such as acupressure or acupuncture (Cheung et al., 2022).

However, this meta-analysis is not without limitations. In particular, the meta-analysis of CAM targeting sleep quality faces a considerable number of constraints. Firstly, publication bias was evident, and the adjusted effect size produced by the trim-and-fill procedure was notably diminished and rendered non-significant. Furthermore, risk of bias also had a significant influence on the effectiveness of CAM, where larger effects were seen in those with a high risk of bias, potentially suggesting inflated effect sizes. These findings corroborate with a previous meta-analysis examining the effects of acupuncture on cancer-related insomnia (Zhang et al., 2022). Although the authors found a large improvement in insomnia, most of the included studies had at least one domain rated at high risk of bias. Thus, the results regarding the effectiveness of CAM in managing insomnia should be considered cautiously. Further rigorous studies are warranted to confirm these findings.

Nonetheless, most of the studies included presented a high risk of bias, which may have affected the reliability of the results. This is likely attributable to the intrinsic nature of the psychological interventions included due to the challenges of blinding and recall biases associated with self-reported outcomes. While the meta-analysis examined diverse cancer types, the participants were over-represented by women and with breast cancer, which is likely to limit the applicability of the results to other lesser studied cancer types. Lastly, the GRADE evaluation showed a “very low” level of confidence in our effect estimates mainly due to considerable level of heterogeneity and inconsistency. This likely stemmed from the design of the current review, such as the inclusion of studies encompassing various cancer types, intervention characteristics, and differing sleep disturbance criteria. Future reviews could enhance the interpretability of intervention outcomes by narrowing the sleep disturbance criteria to specific parameters (e.g., PSQI >5).

## Conclusion

The findings of this study complement previous research in supporting CBT-I as the gold standard and first-line treatment for insomnia and clinically relevant sleep disturbances in cancer patients and survivors, demonstrating sustained effectiveness across different delivery formats. Remote CBT-I programs may be offered as a feasible option for patients to reduce their burden. Although more robust and well-powered studies are needed to confirm the effectiveness of CAM in improving clinical sleep disturbances, evidence suggests its potential in alleviating the physical side effects of treatment. Although effects of multimodal interventions were not assessed in our review, future intervention studies may explore whether integrative approaches, such as combining components from both CAM and CBT-I, may provide a more comprehensive approach for managing sleep disturbances. Lastly, further randomized controlled trials are needed to sufficiently evaluate the effectiveness of other interventions such as mindfulness, exercise-based interventions, herbal medicine, BBT-I, relaxation and pharmacotherapy in managing insomnia or clinical sleep disturbance in cancer populations.

## Data Availability

The raw data supporting the conclusions of this article will be made available by the authors, without undue reservation.
