# Supplementary material for: Interventions for managing clinically relevant sleep disturbances or insomnia in cancer patients and survivors: an up-to-date systematic review and meta-analysis of self-reported sleep disturbance
Source: Front Psychol. 2026 Jun 3;17:1811748. doi: 10.3389/fpsyg.2026.1811748 (PMC13272142; doi:10.3389/fpsyg.2026.1811748)
Supplement: Supplementary file 1 [file Data_Sheet_1.DOCX]

Table of Contents

Supplementary tables 2

Supplementary table 1. Search Strategy 2

Supplementary table 2 4

Table 2a. GRADE rating: Effect of cognitive behavioural therapy for insomnia (CBT-I) 4

Table 2b. GRADE rating: Effect of complementary and alternative medicine (CAM) 6

Table 2c. GRADE rating: Effect of mindfulness-based interventions 7

Table 2d. GRADE rating: Effect of exercise-based interventions 8

Table 2e. GRADE rating: Effect of herbal medicine 9

Table 2f. GRADE rating: Effect of relaxation interventions 10

Table 2g. GRADE rating: Effect of brief behavioural therapy for insomnia (BBT-I) 11

Supplementary figures 12

Supplementary figure 1. Funnel plots for cognitive behavioural therapy for insomnia (CBT-I) on insomnia severity and sleep quality 12

Supplementary figure 2. Funnel plot for complementary and alternative medicine (CAM) on sleep quality 13

Supplementary figure 3. Forest plot of mindfulness-based interventions 14

Supplementary figure 4. Forest plot of exercise-based interventions 15

Supplementary figure 5. Forest plot of herbal medicine 16

Supplementary figure 6. Forest plot of relaxation interventions 17

Supplementary figure 7. Forest plot of brief behavioural therapy for insomnia (BBT-I) 18

Supplementary figure 8. Sensitivity analysis of cognitive behavioural therapy (CBT-I) on insomnia severity with feasibility studies included, with forest plot and funnel plot 19

Supplementary figure 9. Sensitivity analysis of complementary and alternative medicine (CAM) on insomnia severity 20

Supplementary figure 10. Sensitivity analysis of complementary and alternative medicine (CAM) on and sleep quality with feasibility studies included, with forest plot and funnel plot 21

Supplementary figure 11. Sensitivity analysis of brief behavioural therapy for insomnia (BBT-I) on insomnia severity 22

Supplementary material 1 23

| Supplementary tablesSupplementary table 1. Search Strategy | | | | | |
| --- | --- | --- | --- | --- | --- |
|  |  |  |  |  |  |
| **PsychINFO, Web of Science, Embase, CENTRAL** | | | | |  |
| #1 | (sleep):ti,ab |  |  |  |  |
| #2 | (sleep disturbance):ti,ab |  |  |  |  |
| #3 | (sleep quality):ti,ab |  |  |  |  |
| #4 | (insomnia):ti,ab |  |  |  |  |
| #5 | (cancer):ti,ab |  |  |  |  |
| #6 | (neoplasm):ti,ab |  |  |  |  |
| #7 | (intervention):ti,ab |  |  |  |  |
| #8 | (therapy):ti,ab |  |  |  |  |
| #9 | (treatment):ti,ab |  |  |  |  |
| #10 | (programme):ti,ab |  |  |  |  |
| #11 | (program):ti,ab |  |  |  |  |
| #12 | (pharmacological):ti,ab |  |  |  |  |
| #13 | (non-pharmacological):ti,ab |  |  |  |  |
| #14 | (medication):ti,ab |  |  |  |  |
| #15 | (sleep aid):ti,ab |  |  |  |  |
| #16 | (drug):ti,ab |  |  |  |  |
| #17 | (pharmacotherapy):ti,ab |  |  |  |  |
| #18 | (#1 OR #2 OR #3 OR #4) AND (#5 OR #6) AND (#7 OR # 8 OR #9 OR #10 OR #11 OR #12 OR #13 OR #14 OR #15 OR #16 or #17) | | | |  |
| **PubMed** | |  |  |  |  |
| #1 | (sleep):ti,ab OR |  |  |  |  |
|  | (sleep[MeSH Terms]) |  |  |  |  |
| #2 | (sleep disturbance):ti,ab OR |  |  |  |  |
|  | (sleep disturbance[MeSH Terms]) |  |  |  |  |
| #3 | (sleep quality):ti,ab OR |  |  |  |  |
|  | (sleep quality[MeSH Terms]) |  |  |  |  |
| #4 | (insomnia):ti,ab OR |  |  |  |  |
|  | (insomnia[MeSH Terms]) |  |  |  |  |
| #5 | (cancer):ti,ab OR |  |  |  |  |
|  | (cancer[MeSH Terms]) |  |  |  |  |
| #6 | (neoplasm):ti,ab OR |  |  |  |  |
|  | (neoplasm[MeSH Terms]) |  |  |  |  |
| #7 | (intervention):ti,ab OR |  |  |  |  |
|  | (intervention[MeSH Terms]) |  |  |  |  |
| #8 | (therapy):ti,ab OR |  |  |  |  |
|  | (therapy[MeSH Terms]) |  |  |  |  |
| #9 | (treatment):ti,ab OR |  |  |  |  |
|  | (treatment[MeSH Terms]) |  |  |  |  |
| #10 | (programme):ti,ab OR |  |  |  |  |
| #11 | (program):ti,ab |  |  |  |  |
| #12 | (pharmacological):ti,ab OR |  |  |  |  |
|  | (pharmacological[MeSH Terms]) |  |  |  |  |
| #13 | (non-pharmacological):ti,ab OR |  |  |  |  |
| #14 | (medication):ti,ab OR |  |  |  |  |
|  | (medication[MeSH Terms]) |  |  |  |  |
| #15 | (sleep aid):ti,ab OR |  |  |  |  |
|  | sleep aids, pharmaceutical"[MeSH Terms]) |  |  |  |  |
| #16 | (drug):ti,ab |  |  |  |  |
| #17 | (pharmacotherapy):ti,ab OR |  |  |  |  |
|  | (pharmacotherapy[MeSH Terms]) |  |  |  |  |
| #18 | (#1 OR #2 OR #3 OR #4) AND (#5 OR #6) AND (#7 OR # 8 OR #9 OR #10 OR #11 OR #12 OR #13 OR #14 OR #15 OR #16 or #17) | | | | |

## Supplementary table 2

### Table 2a. GRADE rating: Effect of cognitive behavioural therapy for insomnia (CBT-I)

| **Certainty assessment** | | | | | | | **№ of patients** | | **Effect** | | **Certainty** | **Importance** |
| --- | --- | --- | --- | --- | --- | --- | --- | --- | --- | --- | --- | --- |
| **№ of studies** | **Study design** | **Risk of bias** | **Inconsistency** | **Indirectness** | **Imprecision** | **Other considerations** | **CBT-I** | **Comparators** | **Relative (95% CI)** | **Absolute (95% CI)** |  |  |
| **Insomnia Severity (post-treatment)** | | | | | | | | | | | | |
| 14 | randomised trials | serious^a^ | serious^b^ | very serious^c^ | not serious | strong association | 641 | 587 | - | Hedge's g **0.91 higher** (0.49 higher to 1.34 higher) | ⨁◯◯◯ Very low^a,b,c^ | IMPORTANT |
| **Insomnia Severity (short follow-up)** | | | | | | | | | | | | |
| 7 | randomised trials | not serious | serious^d^ | very serious^e^ | not serious | none | 313 | 292 | - | Hedge's g **0.46 higher** (0.05 higher to 0.87 higher) | ⨁◯◯◯ Very low^d,e^ | IMPORTANT |
| **Insomnia Severity (long follow-up)** | | | | | | | | | | | | |
| 4 | randomised trials | not serious | not serious | very serious^e^ | serious^f^ | none | 103 | 89 | - | Hedge's g **0.34 higher** (0.55 lower to 1.24 higher) | ⨁◯◯◯ Very low^e,f^ | IMPORTANT |
| **Sleep Quality (post-treatment)** | | | | | | | | | | | | |
| 11 | randomised trials | serious^a^ | very serious^g^ | very serious^c^ | not serious | none | 413 | 418 | - | Hedge's g **0.57 higher** (0.2 higher to 0.93 higher) | ⨁◯◯◯ Very low^a,c,g^ | IMPORTANT |
| **Sleep Quality (short-follow up)** | | | | | | | | | | | | |
| 6 | randomised trials | not serious | serious^b^ | very serious^e^ | not serious | none | 304 | 285 | - | Hedge's g **0.67 higher** (0.1 higher to 1.24 higher) | ⨁◯◯◯ Very low^b,e^ | IMPORTANT |
| **Sleep quality (long-follow up)** | | | | | | | | | | | | |
| 2 | randomised trials | not serious | not serious | very serious^e,h^ | serious^f^ | none | 59 | 47 | - | Hedge's g **0.07 lower** (4.46 lower to 4.32 higher) | ⨁◯◯◯ Very low^e,f,h^ | IMPORTANT |

**CI:** confidence interval

**Explanations**

a. Majority of studies are high risk of bias

b. I^2^ ≥ 75%

c. Difference in cancer type, treatment status, control type, sleep disturbance requirement

d. I^2^ = 74.3% with wide CI [45.1%; 87.9%]

e. Difference in cancer type, treatment status, control type, sleep disturbance requirement, timepoints

f. Wide 95% CI around the pooled estimate of effect include both little or no effect

g. I^2^ = 73.1%, and subgroup analyses could not explain heterogeneity

h. Treatment status and control type is the same but difference in cancer type, sleep disturbance requirement, timepoints

### Table 2b. GRADE rating: Effect of complementary and alternative medicine (CAM)

| **Certainty assessment** | | | | | | | **№ of patients** | | **Effect** | | **Certainty** | **Importance** |
| --- | --- | --- | --- | --- | --- | --- | --- | --- | --- | --- | --- | --- |
| **№ of studies** | **Study design** | **Risk of bias** | **Inconsistency** | **Indirectness** | **Imprecision** | **Other considerations** | **CAM** | **Comparators** | **Relative (95% CI)** | **Absolute (95% CI)** |  |  |
| **Insomnia Severity (post-treatment)** | | | | | | | | | | | | |
| 3 | randomised trials | serious^a^ | serious^b^ | very serious^c^ | serious^d^ | none | 153 | 141 | - | Hedge's g **0.1 higher** (1.53 lower to 1.73 higher) | ⨁◯◯◯ Very low^a,b,c,d^ | IMPORTANT |
| **Sleep Quality (post-treatment)** | | | | | | | | | | | | |
| 14 | randomised trials | serious^a^ | serious^b^ | very serious^c^ | not serious | publication bias strongly suspected very strong association | 574 | 599 | - | Hedge's g **1.11 higher** (0.43 higher to 1.78 higher) | ⨁◯◯◯ Very low^a,b,c^ | IMPORTANT |
| **Sleep quality (short follow-up)** | | | | | | | | | | | | |
| 5 | randomised trials | not serious | not serious | very serious^e^ | very serious^d,f^ | none | 205 | 208 | - | Hedge's g **0.07 higher** (0.28 lower to 0.41 higher) | ⨁◯◯◯ Very low^d,e,f^ | IMPORTANT |
| **Sleep quality (long follow-up)** | | | | | | | | | | | | |
| 2 | randomised trials | not serious | not serious | very serious^g^ | very serious^d,f^ | none | 88 | 60 | - | Hedge's g **0.02 higher** (0.27 lower to 0.31 higher) | ⨁◯◯◯ Very low^d,f,g^ | IMPORTANT |

**CI:** confidence interval

**Explanations**

a. Majority of studies are high risk of bias

b. I^2^ ≥75%

c. Difference in cancer type, treatment stage, control groups used, sleep disturbance requirement

d. <5 studies, sample size <400

e. Difference in cancer type, treatment stage, control groups used, sleep disturbance requirement, timepoints

f. Wide 95% CI around the pooled estimate of effect include both little or no effect

g. Same cancer type and time points, though the requirement for sleep disturbance and control type is different

### Table 2c. GRADE rating: Effect of mindfulness-based interventions

| **Certainty assessment** | | | | | | | **№ of patients** | | **Effect** | | **Certainty** | **Importance** |
| --- | --- | --- | --- | --- | --- | --- | --- | --- | --- | --- | --- | --- |
| **№ of studies** | **Study design** | **Risk of bias** | **Inconsistency** | **Indirectness** | **Imprecision** | **Other considerations** | **Mindfulness-based interventions** | **Comparators** | **Relative (95% CI)** | **Absolute (95% CI)** |  |  |
| **Insomnia Severity (post-treatment)** | | | | | | | | | | | | |
| 3 | randomised trials | not serious | very serious^a^ | serious^b^ | very serious^c,d^ | none | 128 | 140 | - | Hedge's g **0.26 higher** (2.44 lower to 2.96 higher) | ⨁◯◯◯ Very low^a,b,c,d^ | IMPORTANT |
| **Sleep Quality (post-treatment)** | | | | | | | | | | | | |
| 3 | randomised trials | serious^e^ | very serious^a^ | very serious^f^ | very serious^c,d^ | none | 98 | 127 | - | Hedge's g **0.06 higher** (1.97 lower to 2.08 higher) | ⨁◯◯◯ Very low^a,c,d,e,f^ | IMPORTANT |

**CI:** confidence interval

*Explanations*

a. I^2^ ≥75%

b. Same requirement for sleep disturbance and cancer treatment status, but different cancer type and control type

c. <5 studies, sample size <400

d. Wide 95% CI around the pooled estimate of effect include both little or no effect

e. Majority of studies are high risk of bias

f. Differences in cancer type, treatment status, SD requirement, control type

### Table 2d. GRADE rating: Effect of exercise-based interventions

| **Certainty assessment** | | | | | | | **№ of patients** | | **Effect** | | **Certainty** | **Importance** |
| --- | --- | --- | --- | --- | --- | --- | --- | --- | --- | --- | --- | --- |
| **№ of studies** | **Study design** | **Risk of bias** | **Inconsistency** | **Indirectness** | **Imprecision** | **Other considerations** | **Exercise-based interventions** | **Comparators** | **Relative (95% CI)** | **Absolute (95% CI)** |  |  |
| **Insomnia Severity (post-treatment)** | | | | | | | | | | | | |
| 2 | randomised trials | not serious | not serious | very serious^a^ | very serious^b,c^ | none | 56 | 62 | - | Hedge's g **0.24 lower** (0.57 lower to 0.09 higher) | ⨁◯◯◯ Very low^a,b,c^ | IMPORTANT |
| **Sleep Quality (post-treatment)** | | | | | | | | | | | | |
| 3 | randomised trials | not serious | very serious^d^ | very serious^a^ | very serious^b,c^ | none | 89 | 128 | - | Hedge's g **0.25 higher** (1.56 lower to 2.05 higher) | ⨁◯◯◯ Very low^a,b,c,d^ | IMPORTANT |

**CI:** confidence interval

*Explanations*

a. Taichi used (Irwin et al., 2017) and aerobic exercise used (Mercier et al., 2018) for interventions. Further, while treatment status and control type is the same, cancer type and sleep disturbance requirement is different

b. Wide 95% CI around the pooled estimate of effect include both little or no effect

c. <5 studies, sample size <400

d. I^2^ ≥75%

### Table 2e. GRADE rating: Effect of herbal medicine

| **Certainty assessment** | | | | | | | **№ of patients** | | **Effect** | | **Certainty** | **Importance** |
| --- | --- | --- | --- | --- | --- | --- | --- | --- | --- | --- | --- | --- |
| **№ of studies** | **Study design** | **Risk of bias** | **Inconsistency** | **Indirectness** | **Imprecision** | **Other considerations** | **Herbal Medicine** | **Comparators** | **Relative (95% CI)** | **Absolute (95% CI)** |  |  |
| **Insomnia Severity (post-treatment)** | | | | | | | | | | | | |
| 2 | randomised trials | serious^a^ | not serious | very serious^b^ | very serious^c^ | none | 24 | 24 | - | Hedge's g **0.89 higher** (2.85 lower to 4.64 higher) | ⨁◯◯◯ Very low^a,b,c^ | IMPORTANT |

**CI:** confidence interval

*Explanations*

a. Majority of studies have high risk of bias

b. Cancer type, treatment status is the same but SD requirement and control type is different

c. Wide 95% CI around the pooled estimate of effect include both little or no effect

d. <5 studies, sample size <400

### Table 2f. GRADE rating: Effect of relaxation interventions

| **Certainty assessment** | | | | | | | **№ of patients** | | **Effect** | | **Certainty** | **Importance** |
| --- | --- | --- | --- | --- | --- | --- | --- | --- | --- | --- | --- | --- |
| **№ of studies** | **Study design** | **Risk of bias** | **Inconsistency** | **Indirectness** | **Imprecision** | **Other considerations** | **Relaxation** | **Comparators** | **Relative (95% CI)** | **Absolute (95% CI)** |  |  |
| **Sleep quality (post-treatment)** | | | | | | | | | | | | |
| 4 | randomised trials | very serious^a^ | serious^b^ | serious^c^ | very serious^d,e^ | none | 147 | 159 | - | Hedge's g **1.18 higher** (0.4 lower to 2.75 higher) | ⨁◯◯◯ Very low^a,b,c,d,e^ | IMPORTANT |

**CI:** confidence interval

**Explanations**

a. All studies have high risk of bias

b. I^2^ ≥75%

c. Same treatment status and control type, but different cancer type and sleep disturbance requirement

d. <5 studies, sample size <400

e. Wide 95% CI around the pooled estimate of effect include both little or no effect

### Table 2g. GRADE rating: Effect of brief behavioural therapy for insomnia (BBT-I)

| **Certainty assessment** | | | | | | | **№ of patients** | | **Effect** | | **Certainty** | **Importance** |
| --- | --- | --- | --- | --- | --- | --- | --- | --- | --- | --- | --- | --- |
| **№ of studies** | **Study design** | **Risk of bias** | **Inconsistency** | **Indirectness** | **Imprecision** | **Other considerations** | **BBTI** | **Comparators** | **Relative (95% CI)** | **Absolute (95% CI)** |  |  |
| **Insomnia Severity (post-treatment)** | | | | | | | | | | | | |
| 2 | randomised trials | not serious | serious^a^ | serious^b^ | very serious^c,d^ | none | 78 | 74 | - | Hedge's g **0.95 higher** (6.58 lower to 8.49 higher) | ⨁◯◯◯ Very low^a,b,c,d^ | IMPORTANT |
| **Sleep Quality (post-treatment)** | | | | | | | | | | | | |
| 2 | randomised trials | not serious | not serious | serious^b^ | very serious^c,d^ | none | 77 | 71 | - | Hedge's g **0.48 higher** (1 lower to 1.96 higher) | ⨁◯◯◯ Very low^b,c,d^ | IMPORTANT |

**CI:** confidence interval

**Explanations**

a. I^2^ ≥75%

b. Same control type, but different cancer type, treatment status, and sleep disturbance requirement

c. <5 studies, sample size <400

d. Wide 95% CI around the pooled estimate of effect include both little or no effect

# Supplementary figures

## Supplementary figure 1. Funnel plots for cognitive behavioural therapy for insomnia (CBT-I) on insomnia severity and sleep quality

**Insomnia Severity**


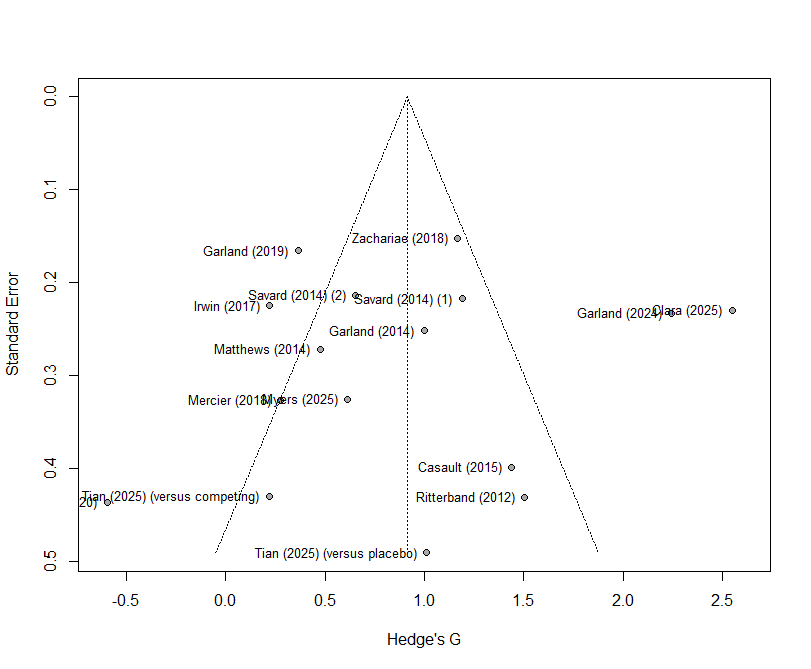


**Sleep Quality**


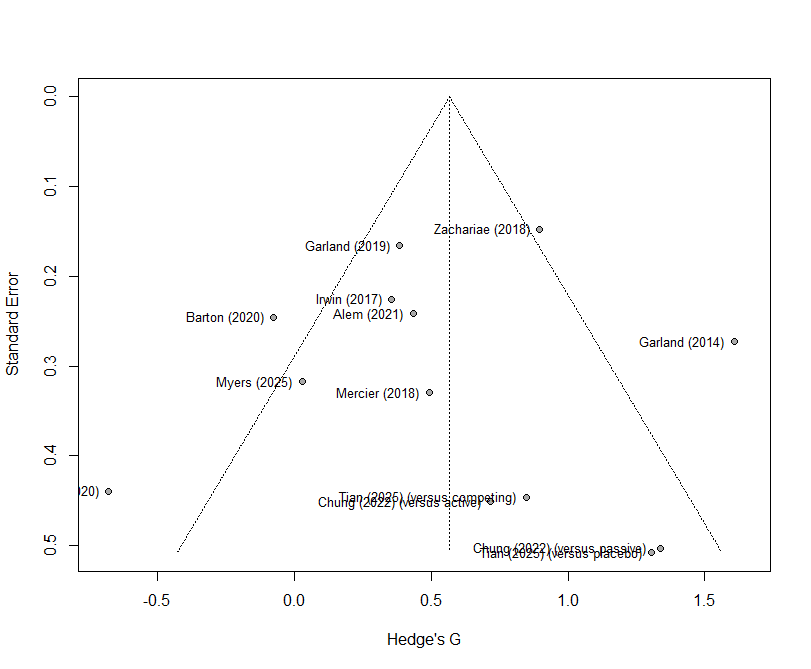


## Supplementary figure 2. Funnel plot for complementary and alternative medicine (CAM) on sleep quality


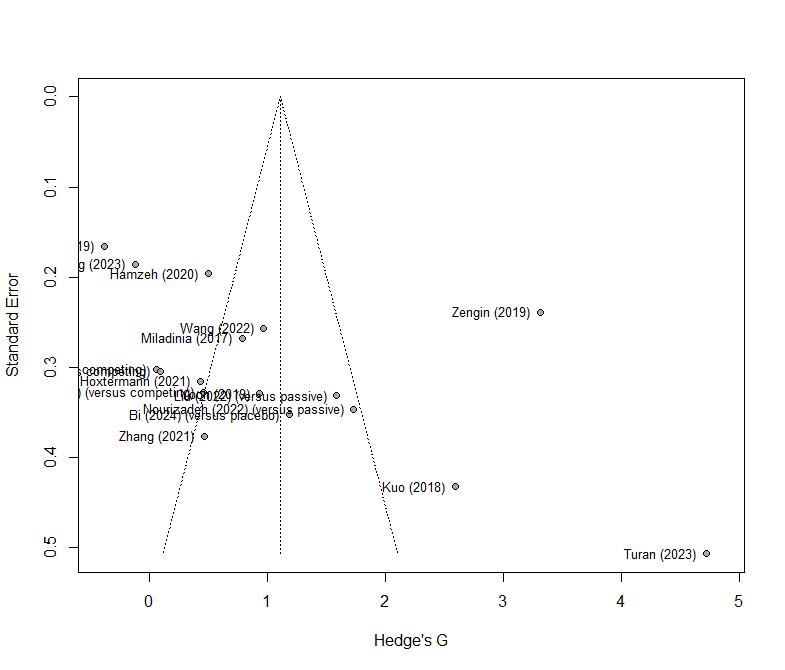


## Supplementary figure 3. Forest plot of mindfulness-based interventions

**Insomnia Severity**


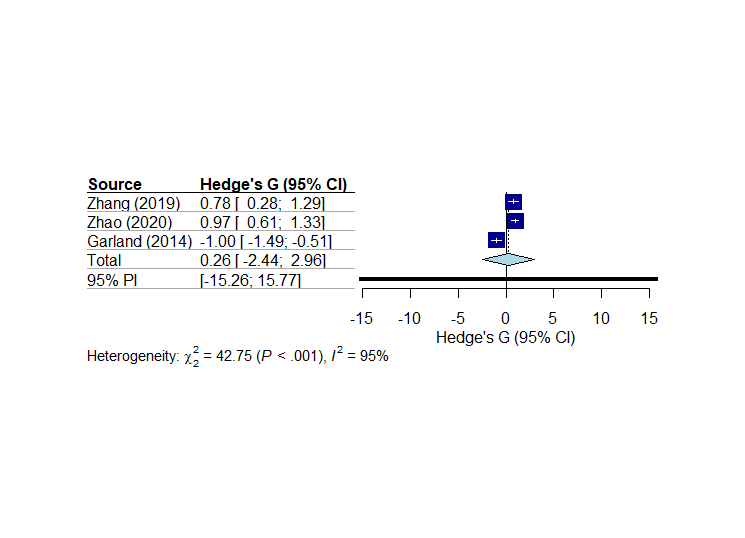


**Sleep Quality**


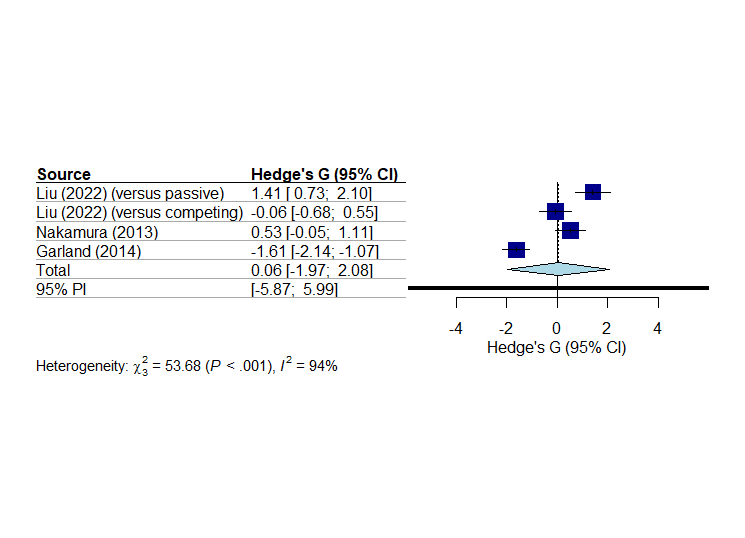


Note: Positive values (right side of the plot) indicate greater improvement with the intervention compared to the comparator.

## Supplementary figure 4. Forest plot of exercise-based interventions

**Insomnia Severity**


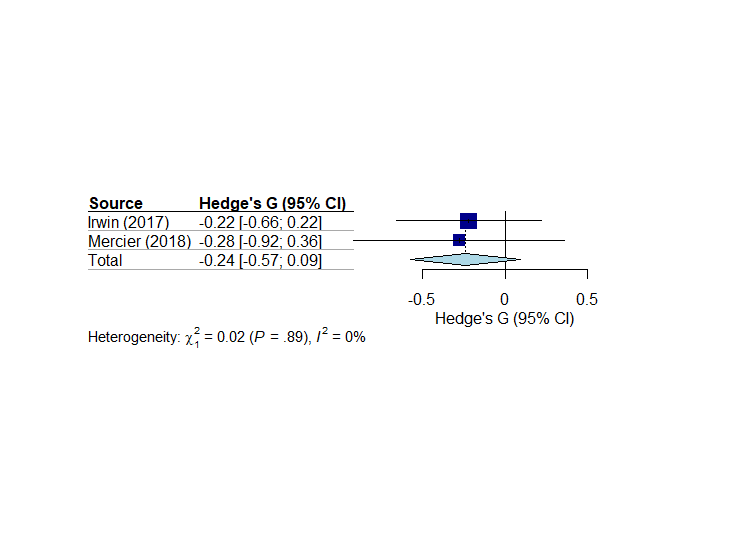


**Sleep Quality**


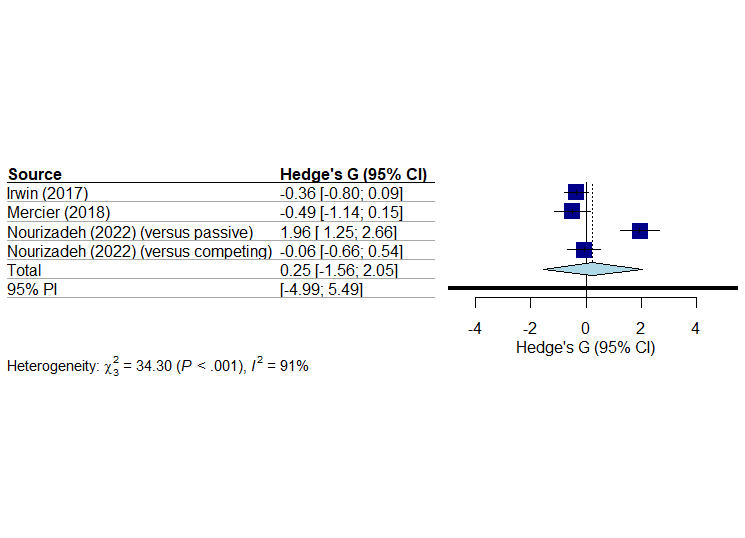


Note: Positive values (right side of the plot) indicate greater improvement with the intervention compared to the comparator.

## Supplementary figure 5. Forest plot of herbal medicine

**Insomnia Severity**


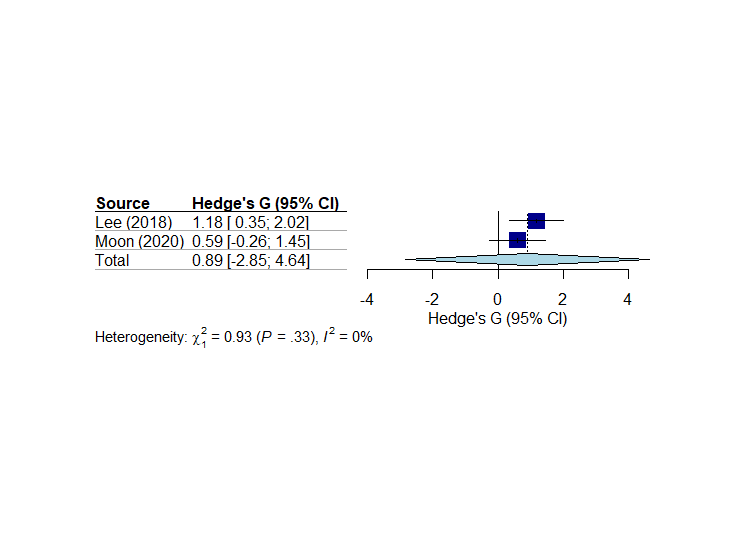


Note: Positive values (right side of the plot) indicate greater improvement with the intervention compared to the comparator.

## Supplementary figure 6. Forest plot of relaxation interventions

**Sleep Quality**


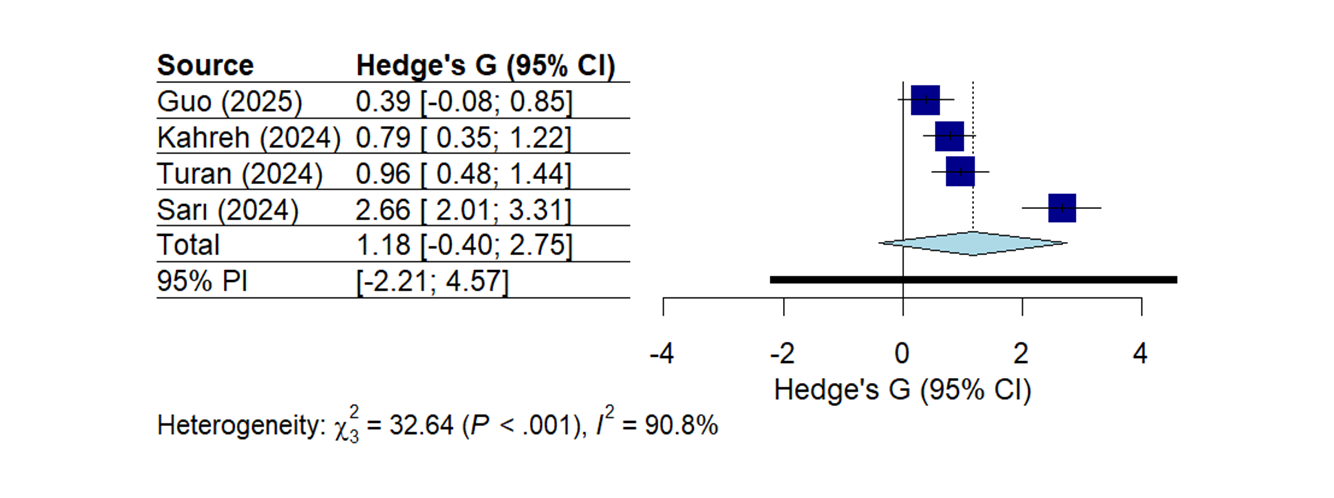


Note: Positive values (right side of the plot) indicate greater improvement with the intervention compared to the comparator.

## Supplementary figure 7. Forest plot of brief behavioural therapy for insomnia (BBT-I)

**Insomnia Severity**


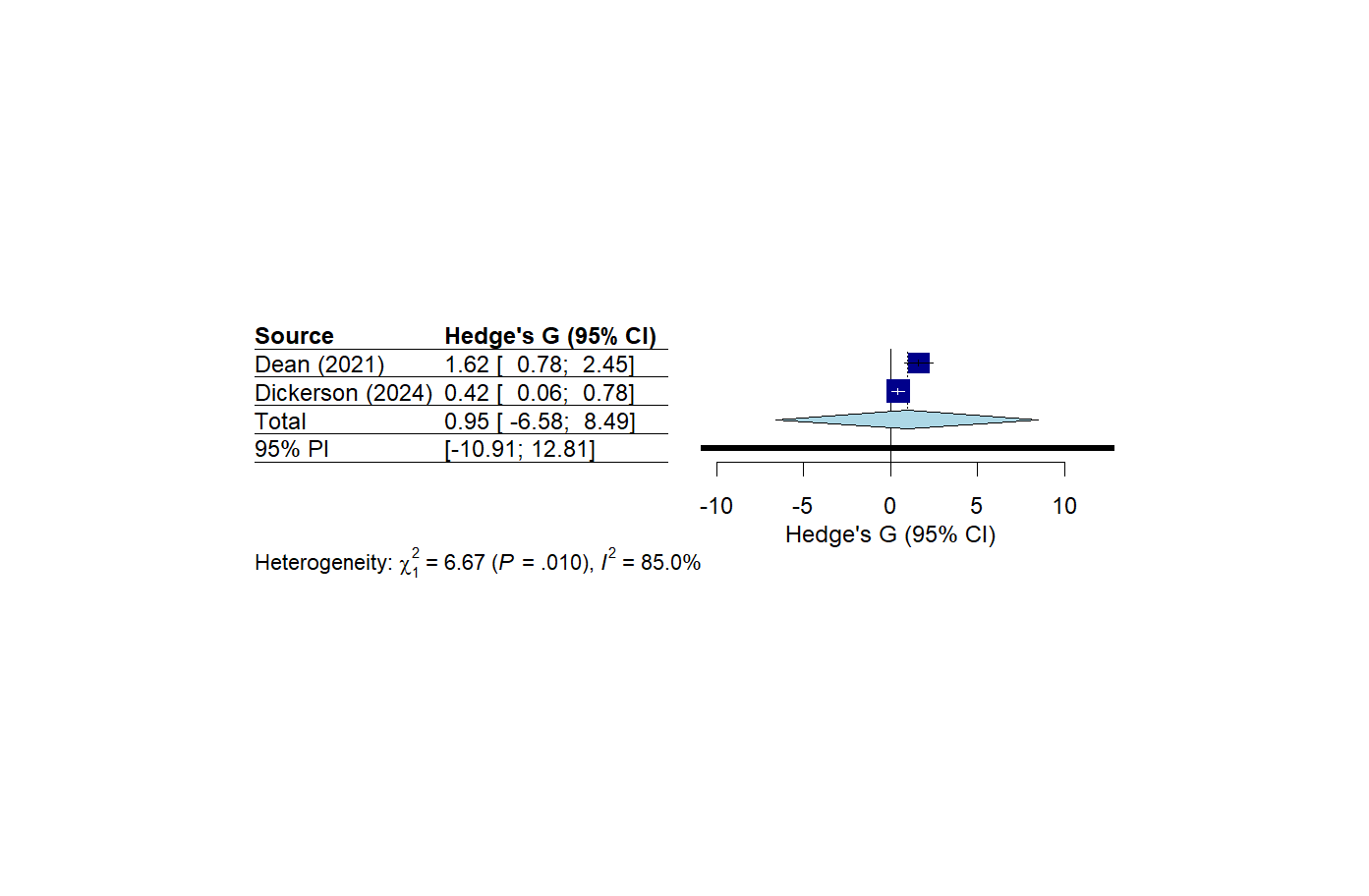


**Sleep Quality**


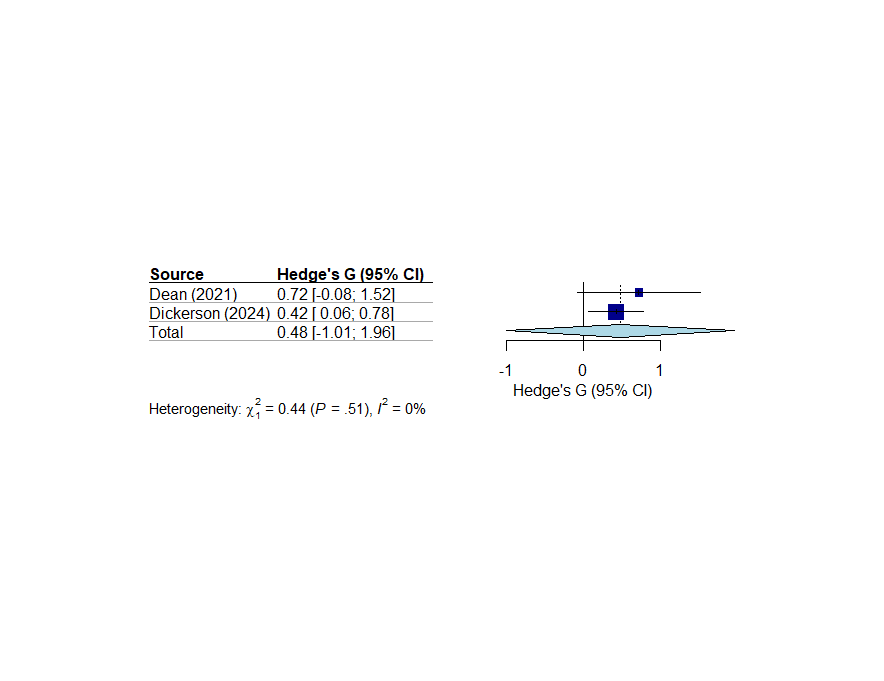


Note: Positive values (right side of the plot) indicate greater improvement with the intervention compared to the comparator.

## Supplementary figure 8. Sensitivity analysis of cognitive behavioural therapy (CBT-I) on insomnia severity with feasibility studies included, with forest plot and funnel plot


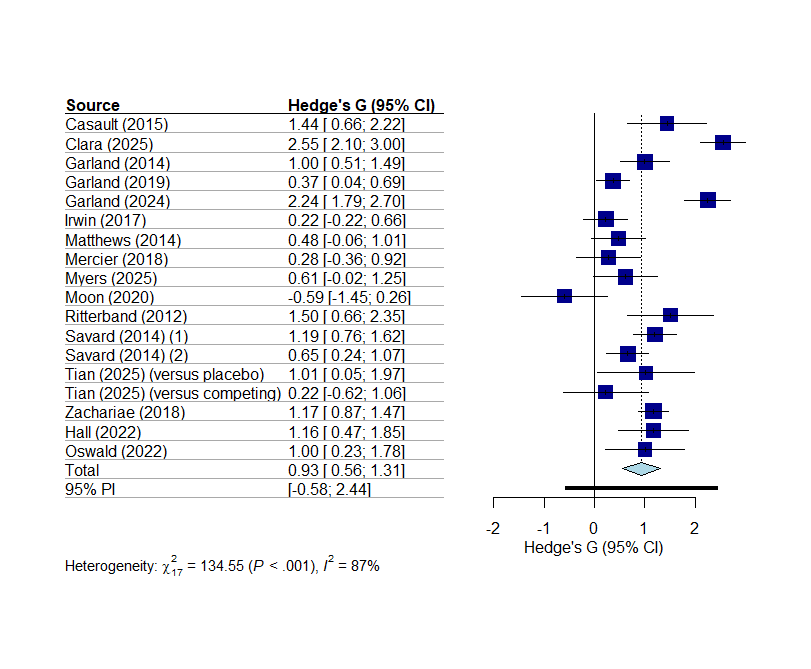


Note: Positive values (right side of the forest plot) indicate greater improvement with the intervention compared to the comparator. Hall (2022) and Oswald (2022) were excluded as feasibility studies during the selection process.


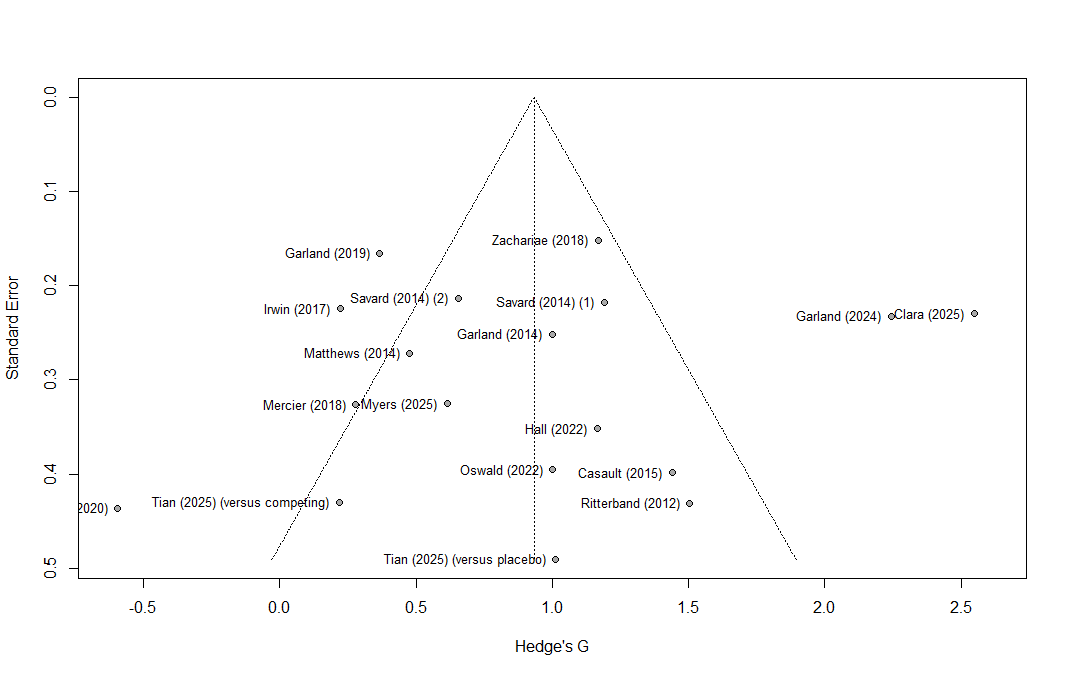


## Supplementary figure 9. Sensitivity analysis of complementary and alternative medicine (CAM) on insomnia severity


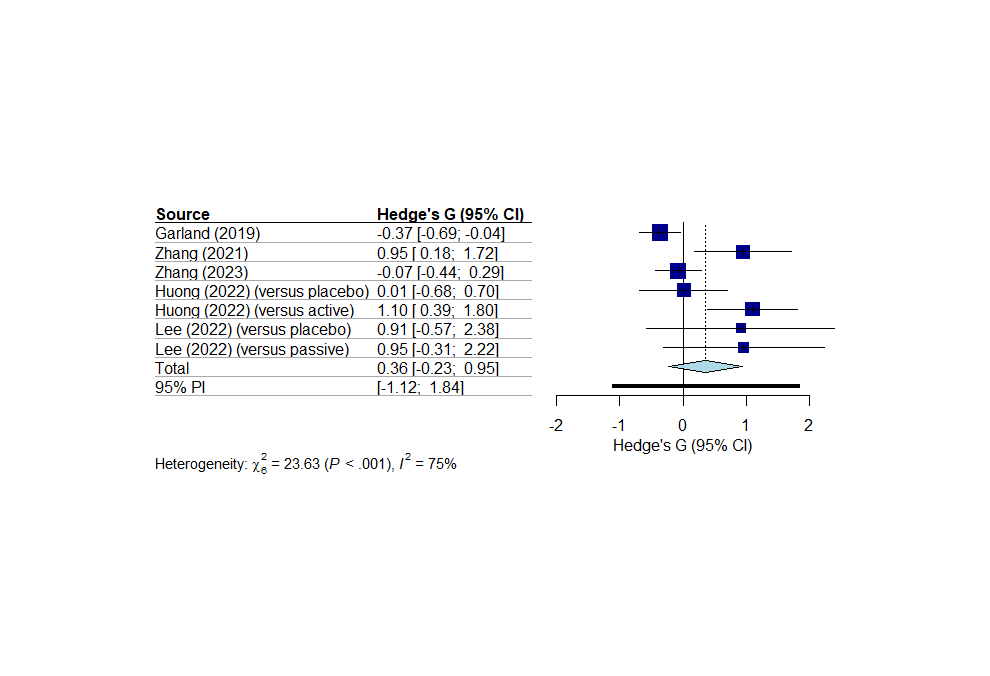


Note: Positive values (right side of the plot) indicate greater improvement with the intervention compared to the comparator. Huong (2022) and Lee (2022) were excluded as feasibility studies during the selection process.

## Supplementary figure 10. Sensitivity analysis of complementary and alternative medicine (CAM) on and sleep quality with feasibility studies included, with forest plot and funnel plot


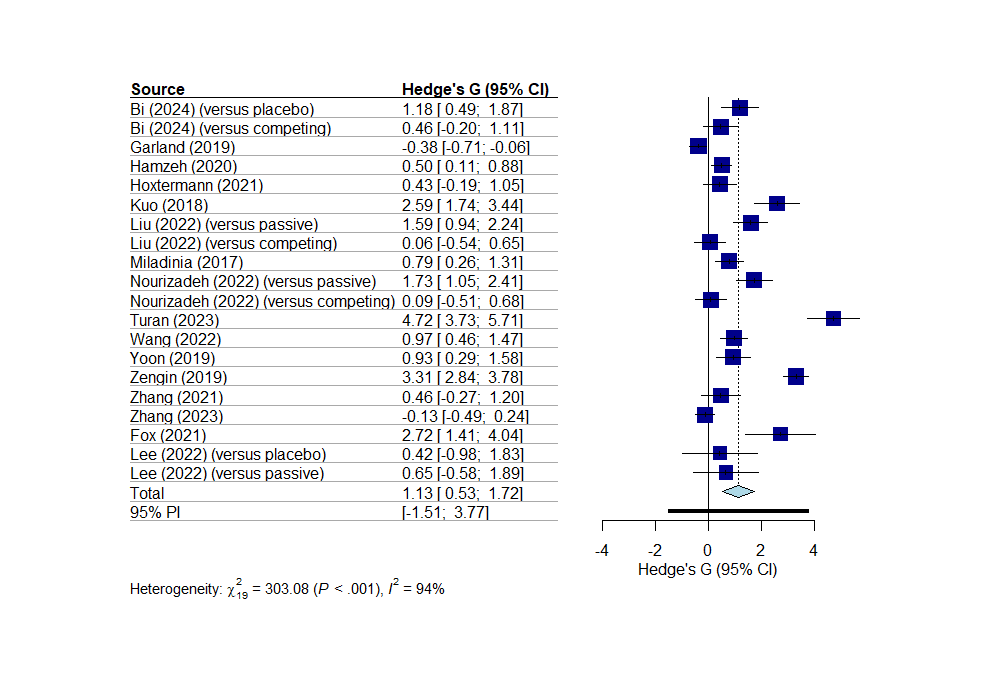


Note: Positive values (right side of the forest plot) indicate greater improvement with the intervention compared to the comparator. Fox (2021) and Lee (2022) were excluded as feasibility studies during the selection process.


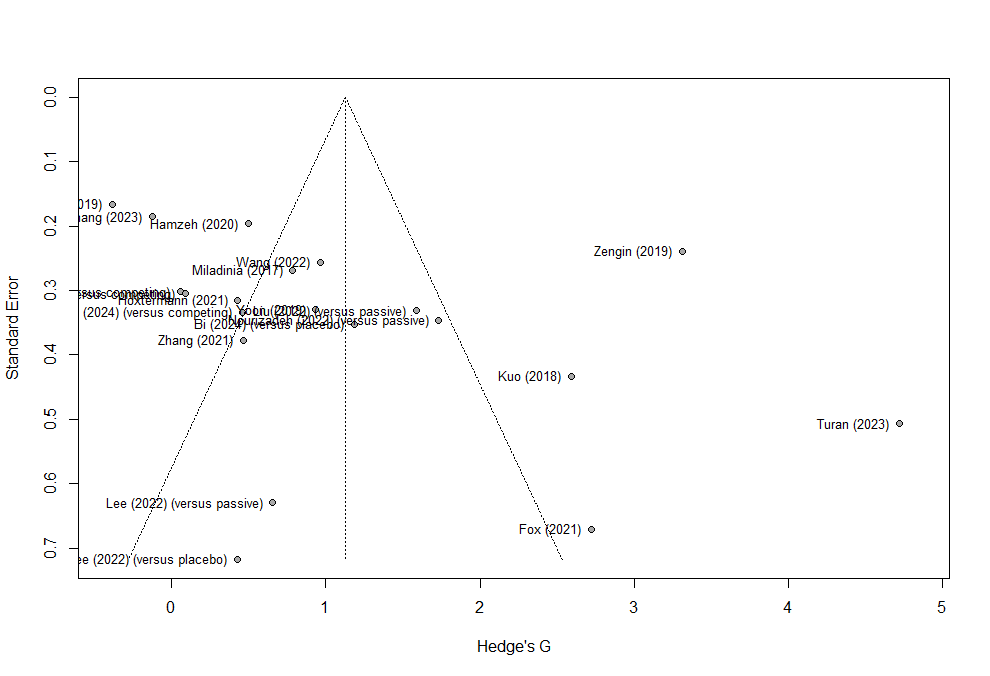


## Supplementary figure 11. Sensitivity analysis of brief behavioural therapy for insomnia (BBT-I) on insomnia severity


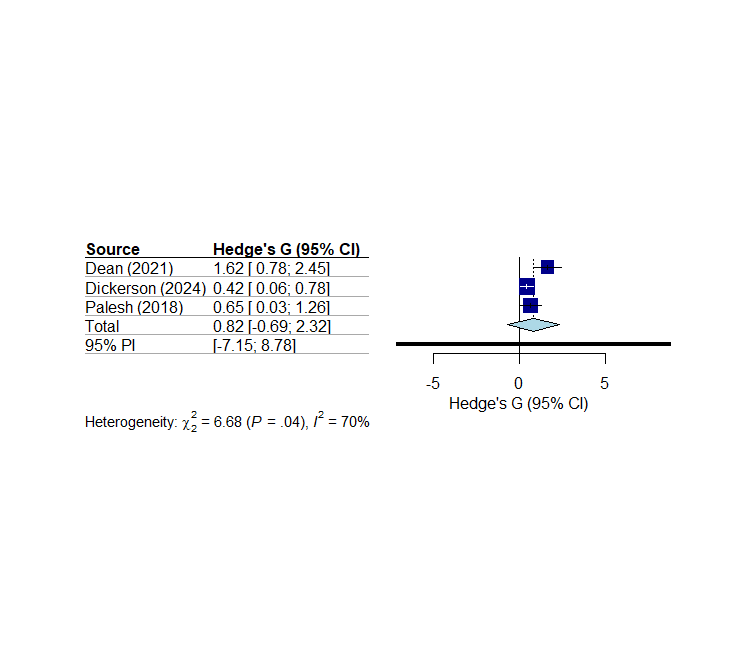


Note: Positive values (right side of the plot) indicate greater improvement with the intervention compared to the comparator. Palesh (2018) was excluded as feasibility studies during the selection process.

# Supplementary material 1

All analyses cited below were conducted in R.

Meta-analysis & forest plot:

cbti_isi_result<-metagen(g,se,data=cbti_isi, studlab=paste(study), comb.fixed=FALSE,comb.random=TRUE,method.tau="SJ",hakn=TRUE,prediction=TRUE,sm="Hedge's G", print.byvar = TRUE, summary = TRUE)

forest(cbti_isi_result, layout= "JAMA", text.predict="95% PI", col.predict="black")

Egger’s test & funnel plot:

metabias(cbti_isi_result)

funnel(cbti_isi_result, studlab = TRUE)

Duval and Tweedie Trim-Fill procedure:

tf_CAM_psqi_result<-trimfill(CAM_psqi_result)

Subgroup analysis:

risk_cbti_isi_result<-metagen(g,se,data=cbti_isi, byvar=bias, studlab=paste(study), comb.fixed=FALSE,comb.random=TRUE,method.tau="SJ",hakn=TRUE,prediction=TRUE,sm="Hedge's G", print.byvar = TRUE, summary = TRUE)
